# Supplementary material for: Non-vesicular phosphatidylinositol transfer plays critical roles in defining organelle lipid composition
Source: EMBO J. 2024 Apr 16;43(10):6. doi: 10.1038/s44318-024-00096-3 (PMC11099152; doi:10.1038/s44318-024-00096-3)
Supplement: Supplementary file 1 — Table EV1 [file 44318_2024_96_MOESM1_ESM.docx]

**Table EV1. Primers used in this study.**

| **Construct name** |  | **Primer sequences 5’ to 3’** |
| --- | --- | --- |
| Human PITPNA | F | GCGAAGCGACATGGTGCTGCTC |
|  | R | GGAAAGGCGGCTTTAGTCATCTG |
| EGFP-PITPNA | F | AAAGAATTCGAGCGACATGGTGCTGCTCAAG |
|  | R | CGGTGGATCCTTAGTCATCTGCTGTCATTCC |
| EGFP-PITPNA- Δ5 | F | ATATGAATTCAATGGTGCTGCTCAAGGAGTATCGAG |
|  | R | ATATGGATCCCTATCCTTTCACTGGGTCCTTTTGTCTCATTTC |
| EGFP-PITPNB | F | ACAAAGCTTGGAAGATGGTGCTGATCAAG |
|  | R | ACAGGATCCTCATCTAGACATCAGCAGCC |
| EGFP-PITPNB- Δ6 | F | ATATCAAGCTTGGAAGGGTGGAGCTG |
|  | R | ATATGGATCCCTAGCCTCGAACGGAACCCCTCTTAC |
| EGFP-PITPNA  (FV/AA) | F | CAGAGCAAAGTACCCACGGCTGCTCGAATGCTGGCCCCAGAG |
|  | R | CTCTGGGGCCAGCATTCGAGCAGCCGTGGGTACTTTGCTCTG |
| EGFP-PITPNA  (WW/AA) | F | TCAAGGCGGCGGGCCTGCAGAAC |
|  | R | AGGCCCGCCGCCTTGAACTTGACG |
| PITPNA  (C94S) | F | CCTACTCCAGAACCGTTATTACAAATGAG |
|  | R | TTCTGGAGTAGGGGTAAGCATTCC |
| PITPNA  (C94A) | F | CCTACTCCAGAACCGTTATTACAAATGAG |
|  | R | TTCTGGCGTAGGGGTAAGCATTCC |
| PITPNA  (C94T) | F | CCTACACCAGAACCGTTATTACAAATGAG |
|  | R | TTCTGGTGTAGGGGTAAGCATTCC |
| PITPNA  (T58E) | F | TGAGAAAGGCCAGTACGAACACAAGATCTACC |
|  | R | CAGGTGGTAGATCTTGTGTTCGTACTGGC |
| PITPNB  (C94S) | F | TACCCCTACTCTAGAACAATTGTAACGAATG |
|  | R | CGTTACAATTGTTCTAGAGTAGGGGTACGC |
| PITPNB  (FV/AA) | F | CCTGCAGCCGCGAGGATGATTGCTCCC G |
|  | R | CATCCTCGCGGCTGCAGGCACTTTGCTCTTTAGG |
| PITPNB  (WW/AA) | F | GGTGACCATCAAATTCAAGGCGGCGGGACTGCAAAGCAAAGTAG |
|  | R | CTACTTTGCTTTGCAGTCCCGCCGCCTTGAATTTGATGGTCACC |
| PITPNB  (T58A) | F | GGAGAAAAGGGACAGTATGCGCACAAAATTTATCACC |
|  | R | GGTGATAAATTTTGTGCGCATACTGTCCCTTTTCTCC |
| PITPNB  (T58E) | F | GGAGAAAAGGGACAGTATGAGCACAAAATTTATCACA |
|  | R | GGTGATAAATTTTGTGCTCATACTGTCCCTTTTCTCC |
| EGFP-GOLPH3 | F | AAAATCTCGAGCGACCTCGCTGACCCAGCGCAG |
|  | R | ATATGAATTCGAGAATGGTTCACCCCGAGCAGAG |
