# Supplementary figures and images for: Non-vesicular phosphatidylinositol transfer plays critical roles in defining organelle lipid composition
Source: EMBO J. 2024 Apr 16;43(10):6. doi: 10.1038/s44318-024-00096-3 (PMC11099152; doi:10.1038/s44318-024-00096-3)

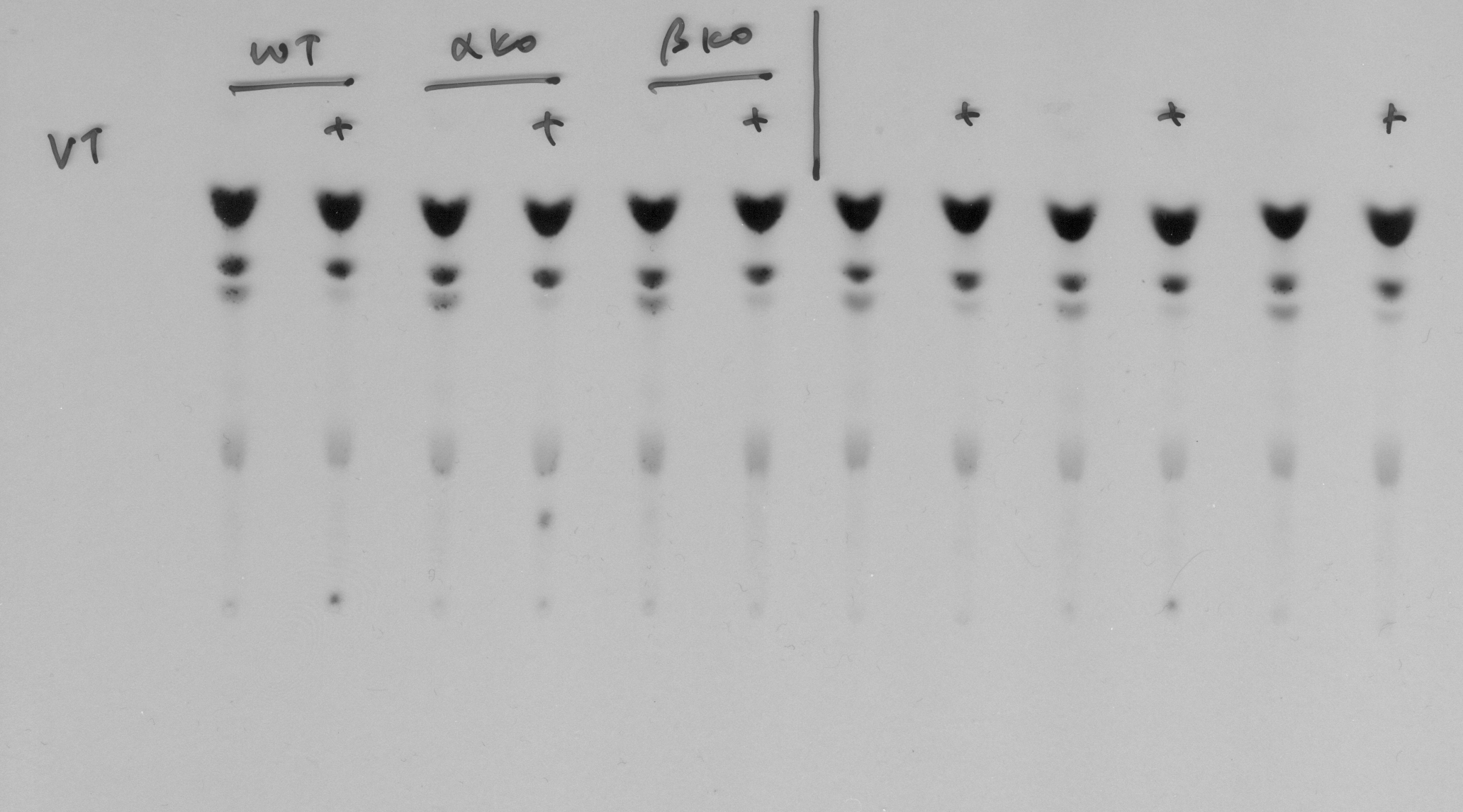

Supplement: Supplementary file 2 — Source data Fig. 1 [file 44318_2024_96_MOESM2_ESM.zip › Figure 1/1A/Figure1A-PI labeling-TLC image.tif]

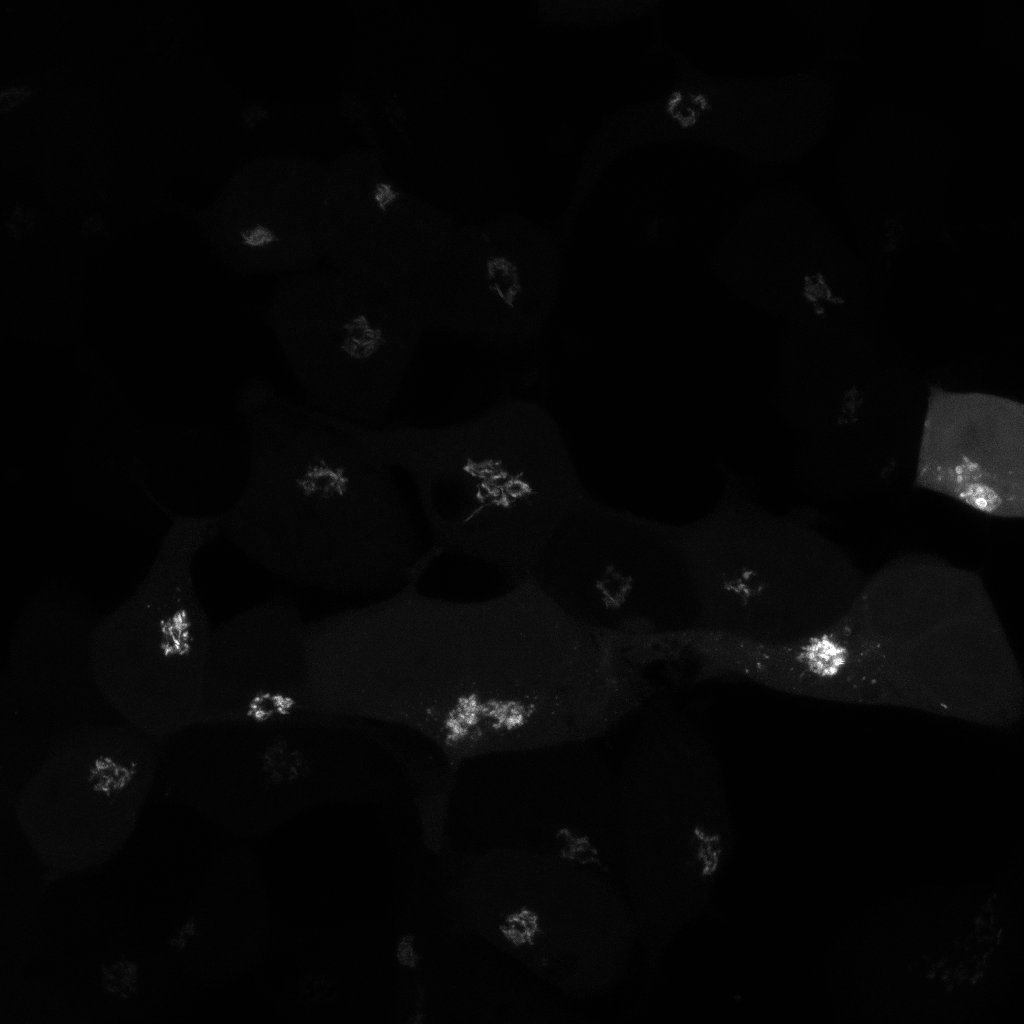

Supplement: Supplementary file 3 — Source data Fig. 2 [file 44318_2024_96_MOESM3_ESM.zip › Figure 2/2A/2A-FAPP1PH-VT 0 min.Jpeg]

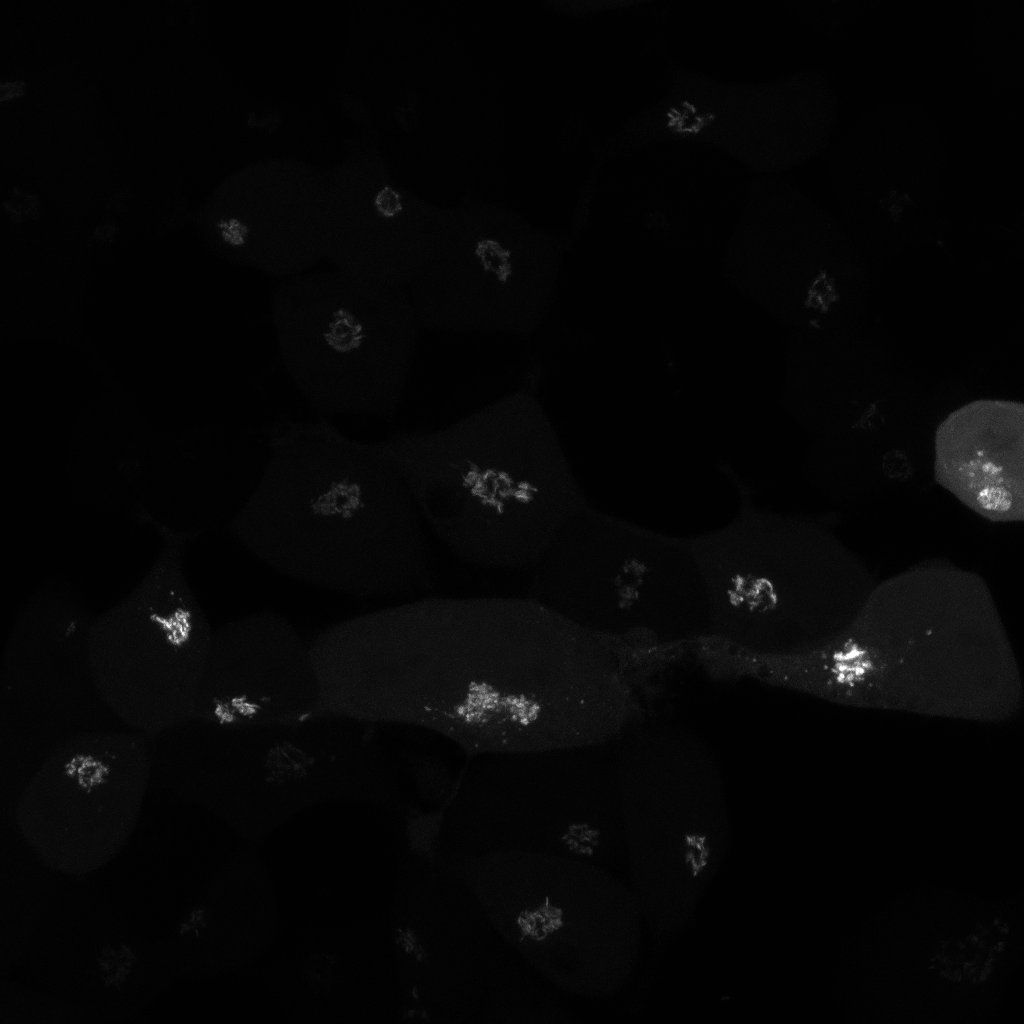

Supplement: Supplementary file 3 — Source data Fig. 2 [file 44318_2024_96_MOESM3_ESM.zip › Figure 2/2A/2A-FAPP1PH-VT30 min.Jpeg]

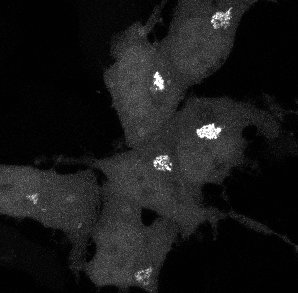

Supplement: Supplementary file 3 — Source data Fig. 2 [file 44318_2024_96_MOESM3_ESM.zip › Figure 2/2F/2F-FAPP2PH-0min.jpeg]

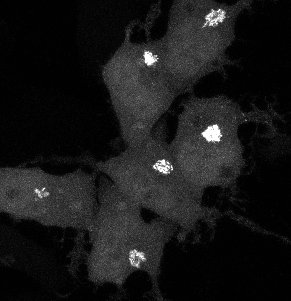

Supplement: Supplementary file 3 — Source data Fig. 2 [file 44318_2024_96_MOESM3_ESM.zip › Figure 2/2F/2F-FAPP2PH-VT30min.Jpeg]

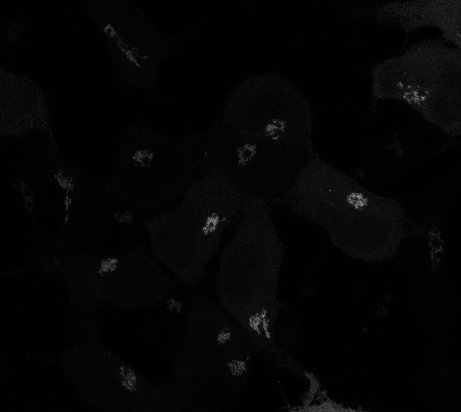

Supplement: Supplementary file 3 — Source data Fig. 2 [file 44318_2024_96_MOESM3_ESM.zip › Figure 2/2H/2H-Golph3-0min.jpeg]

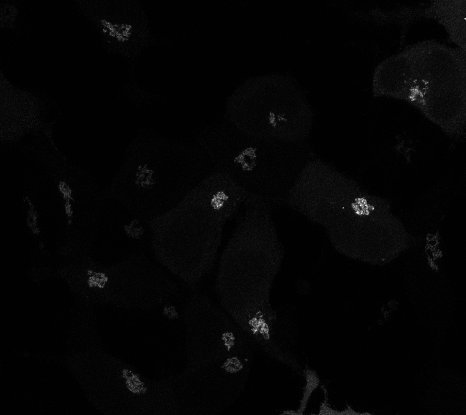

Supplement: Supplementary file 3 — Source data Fig. 2 [file 44318_2024_96_MOESM3_ESM.zip › Figure 2/2H/2H-Golph3-VT30min.jpeg]

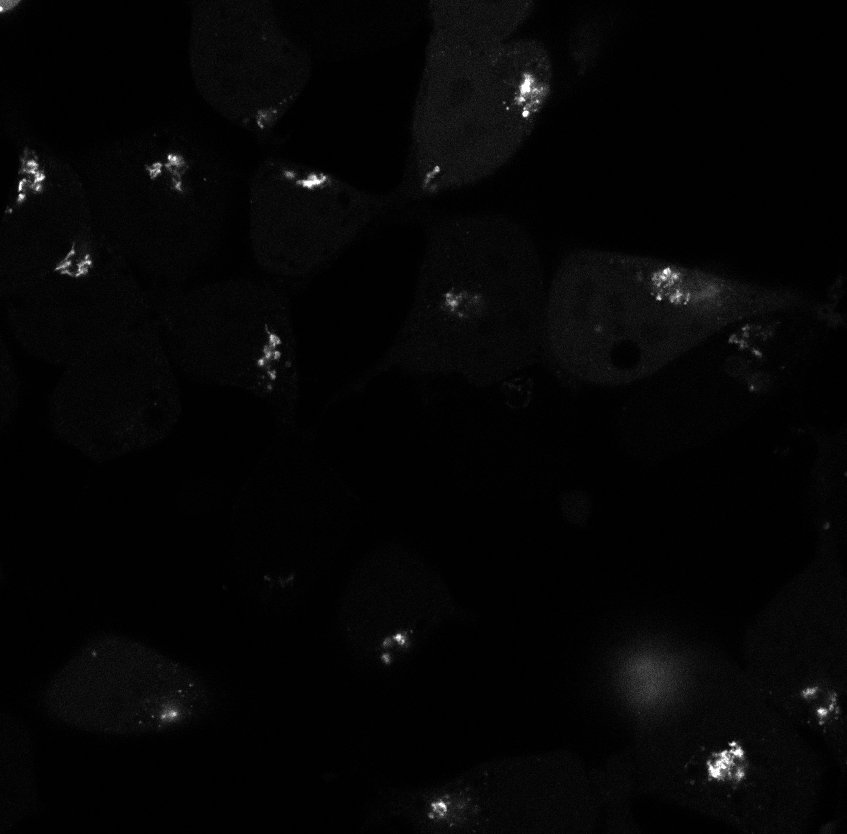

Supplement: Supplementary file 3 — Source data Fig. 2 [file 44318_2024_96_MOESM3_ESM.zip › Figure 2/2J/2J-CERTPH-0min.jpeg]

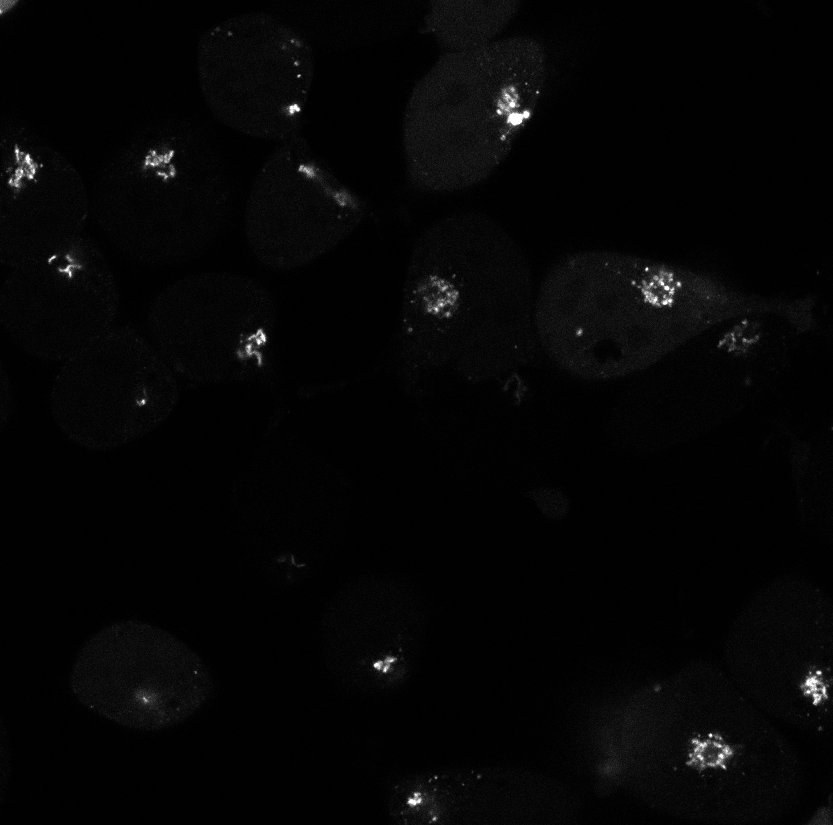

Supplement: Supplementary file 3 — Source data Fig. 2 [file 44318_2024_96_MOESM3_ESM.zip › Figure 2/2J/2J-CERTPH-VT30min.jpeg]

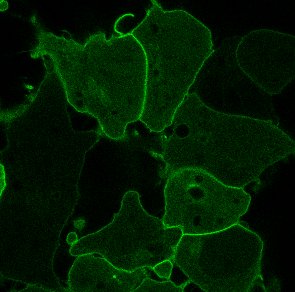

Supplement: Supplementary file 6 — Source data Fig. 5 [file 44318_2024_96_MOESM6_ESM.zip › Figure 5/5A/5A-AngII 5 min.jpg]

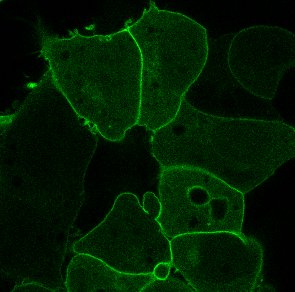

Supplement: Supplementary file 6 — Source data Fig. 5 [file 44318_2024_96_MOESM6_ESM.zip › Figure 5/5A/5A-AngII+DGKi.jpg]

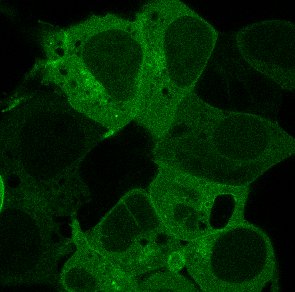

Supplement: Supplementary file 6 — Source data Fig. 5 [file 44318_2024_96_MOESM6_ESM.zip › Figure 5/5A/5A-control.jpg]

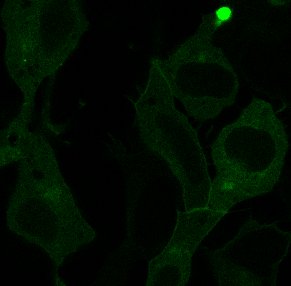

Supplement: Supplementary file 6 — Source data Fig. 5 [file 44318_2024_96_MOESM6_ESM.zip › Figure 5/5C/5C-AngII 5 min.jpg]

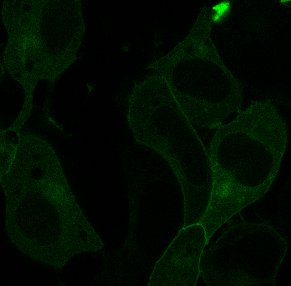

Supplement: Supplementary file 6 — Source data Fig. 5 [file 44318_2024_96_MOESM6_ESM.zip › Figure 5/5C/5C-AngII+DGKi.jpg]

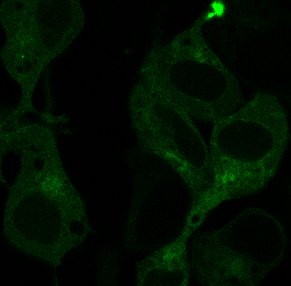

Supplement: Supplementary file 6 — Source data Fig. 5 [file 44318_2024_96_MOESM6_ESM.zip › Figure 5/5C/5C-control.jpg]

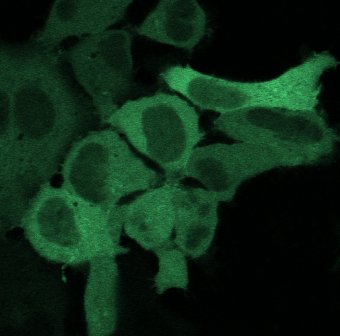

Supplement: Supplementary file 6 — Source data Fig. 5 [file 44318_2024_96_MOESM6_ESM.zip › Figure 5/5F/5F-control.jpg]

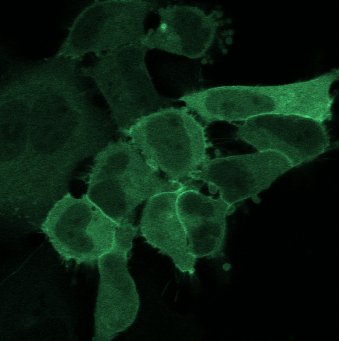

Supplement: Supplementary file 6 — Source data Fig. 5 [file 44318_2024_96_MOESM6_ESM.zip › Figure 5/5F/5F-PMA.jpg]

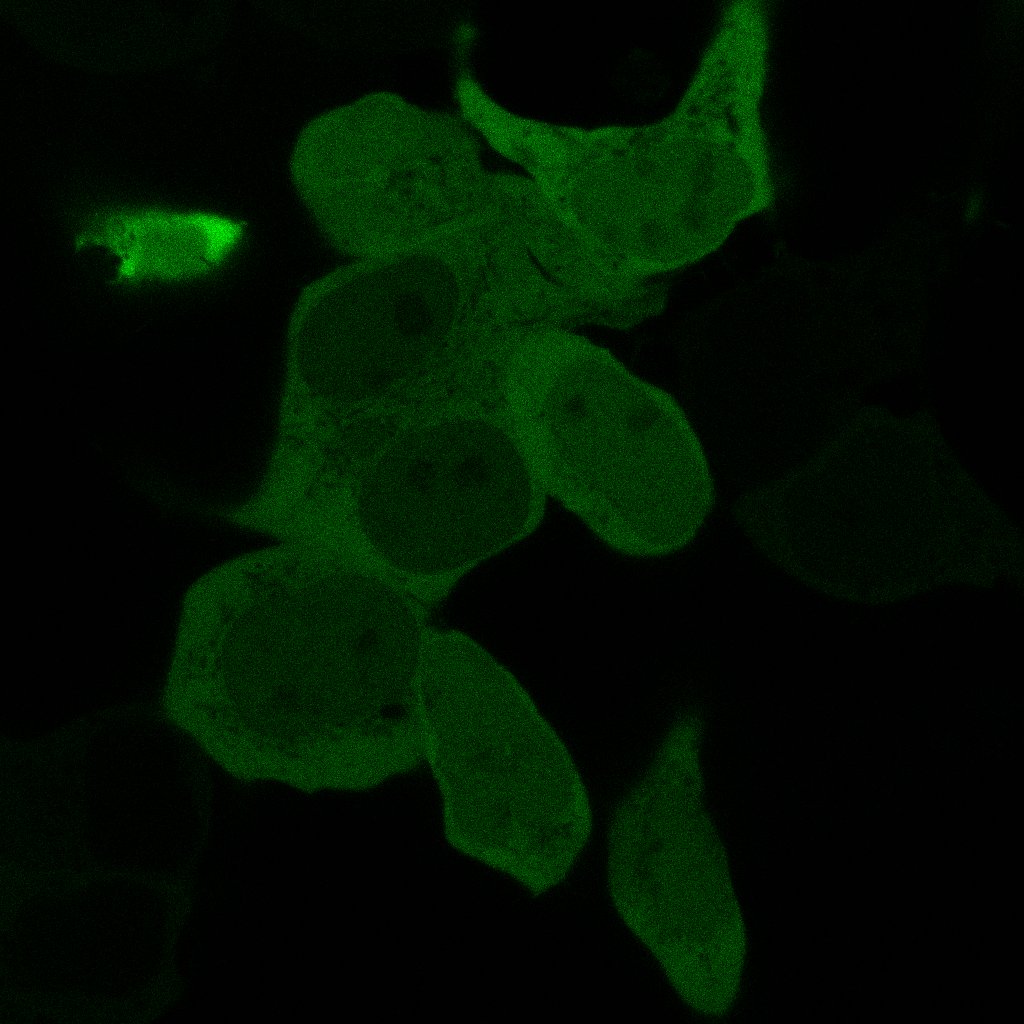

Supplement: Supplementary file 6 — Source data Fig. 5 [file 44318_2024_96_MOESM6_ESM.zip › Figure 5/5H/5H-lower-AngII 5 min.jpg]

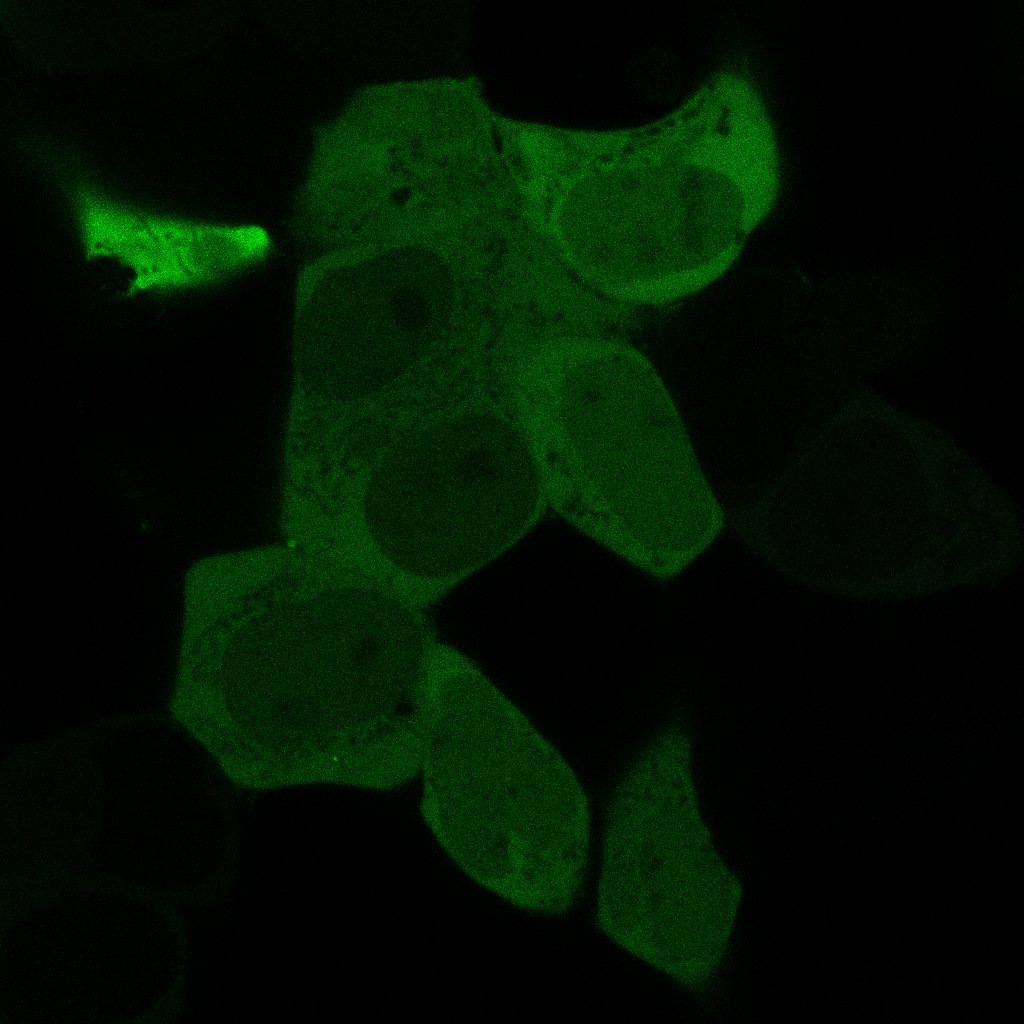

Supplement: Supplementary file 6 — Source data Fig. 5 [file 44318_2024_96_MOESM6_ESM.zip › Figure 5/5H/5H-lower-control.jpg]

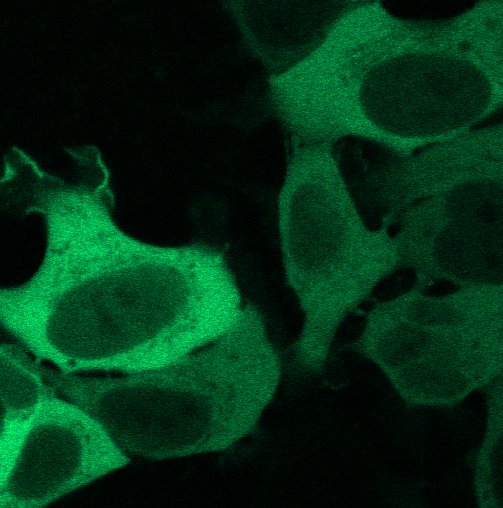

Supplement: Supplementary file 6 — Source data Fig. 5 [file 44318_2024_96_MOESM6_ESM.zip › Figure 5/5H/5H-upper-AngII 5 min.jpg]

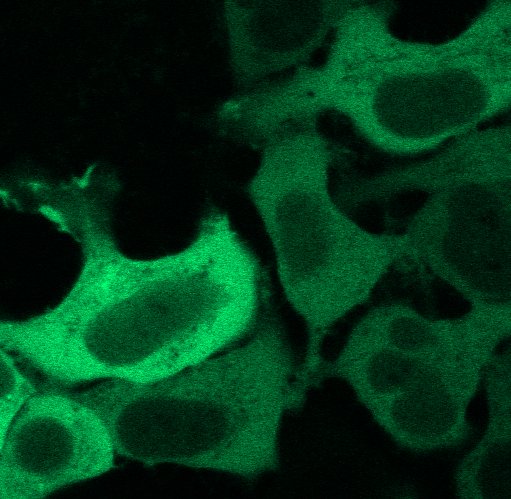

Supplement: Supplementary file 6 — Source data Fig. 5 [file 44318_2024_96_MOESM6_ESM.zip › Figure 5/5H/5H-upper-control.jpg]

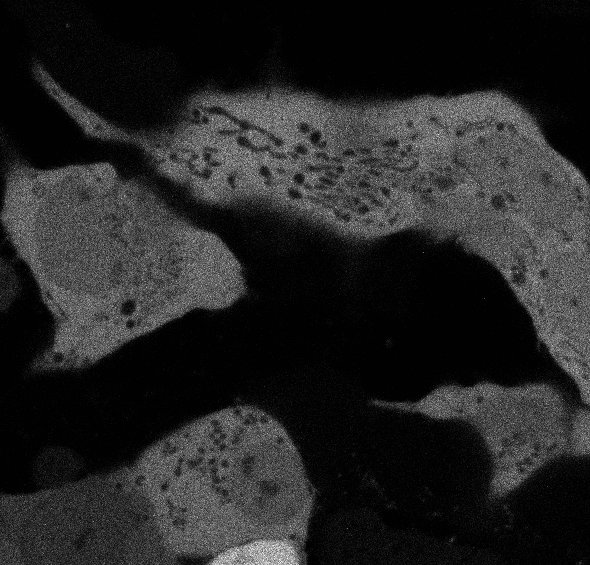

Supplement: Supplementary file 7 — Source data Fig. 6 [file 44318_2024_96_MOESM7_ESM.zip › Figure 6/6B/6B-PITPNB-control before VT.jpg]

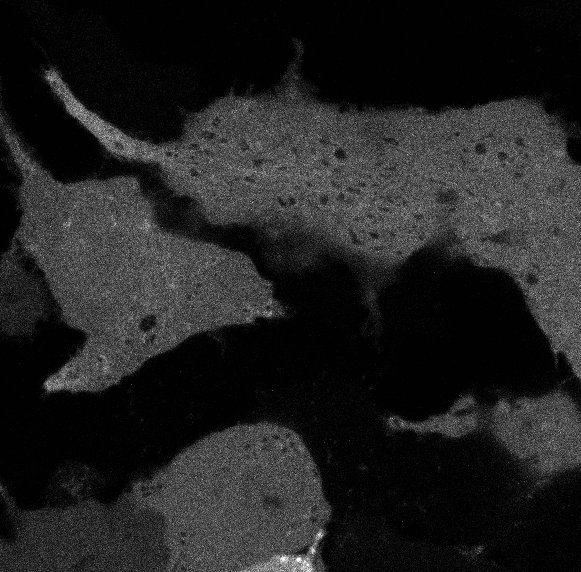

Supplement: Supplementary file 7 — Source data Fig. 6 [file 44318_2024_96_MOESM7_ESM.zip › Figure 6/6B/6B-PITPNB-VT 10 min.jpg]

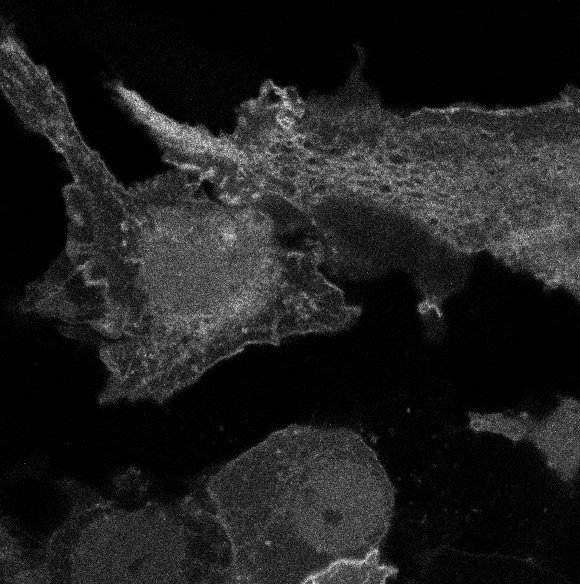

Supplement: Supplementary file 7 — Source data Fig. 6 [file 44318_2024_96_MOESM7_ESM.zip › Figure 6/6B/6B-PITPNB-VT+AngII 5 min.jpg]

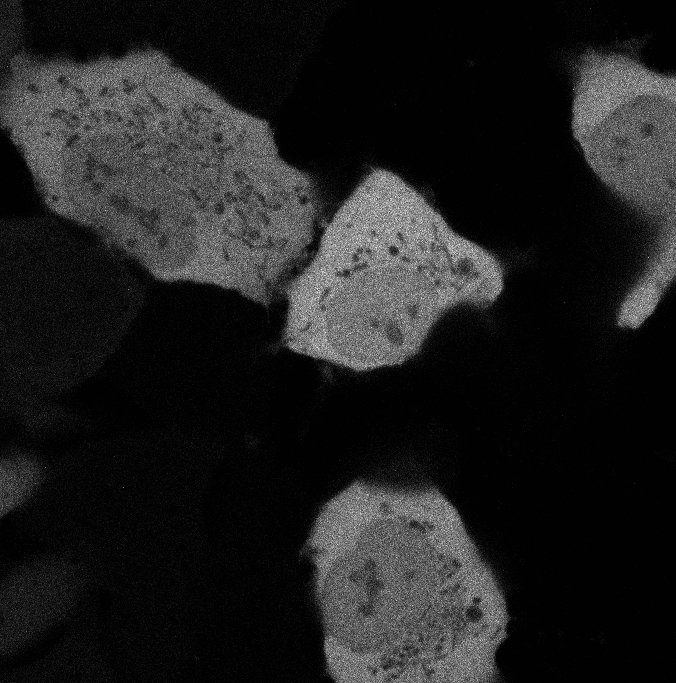

Supplement: Supplementary file 7 — Source data Fig. 6 [file 44318_2024_96_MOESM7_ESM.zip › Figure 6/6C/6C-PITPNB-WWAA-control before VT.jpg]

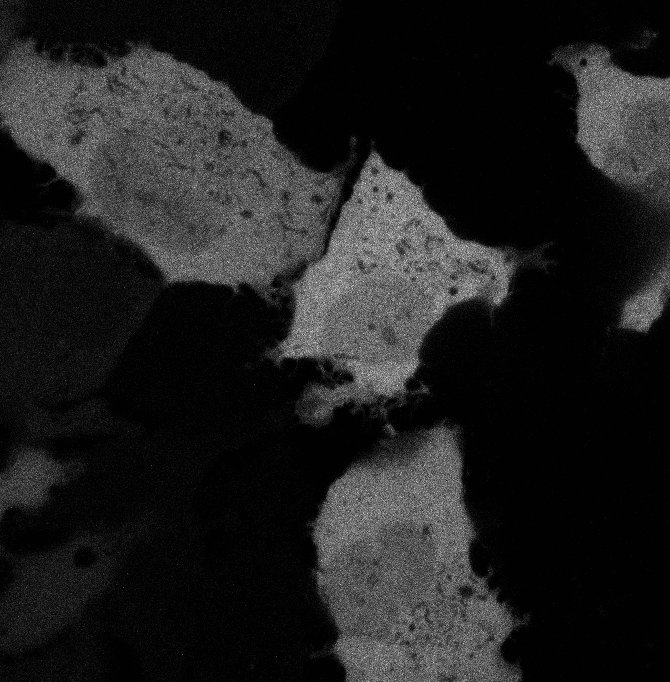

Supplement: Supplementary file 7 — Source data Fig. 6 [file 44318_2024_96_MOESM7_ESM.zip › Figure 6/6C/6C-PITPNB-WWAA-VT 10 min.jpg]

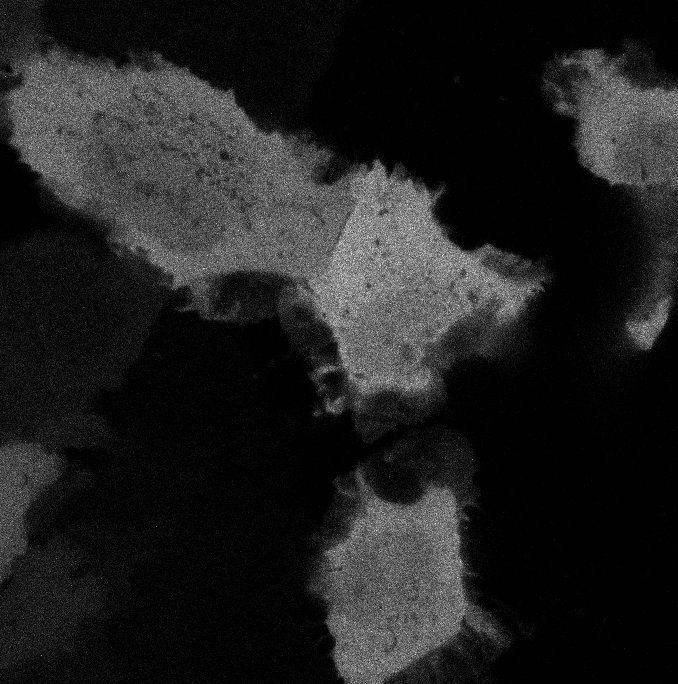

Supplement: Supplementary file 7 — Source data Fig. 6 [file 44318_2024_96_MOESM7_ESM.zip › Figure 6/6C/6C-PITPNB-WWAA-VT+AngII 5 min.jpg]

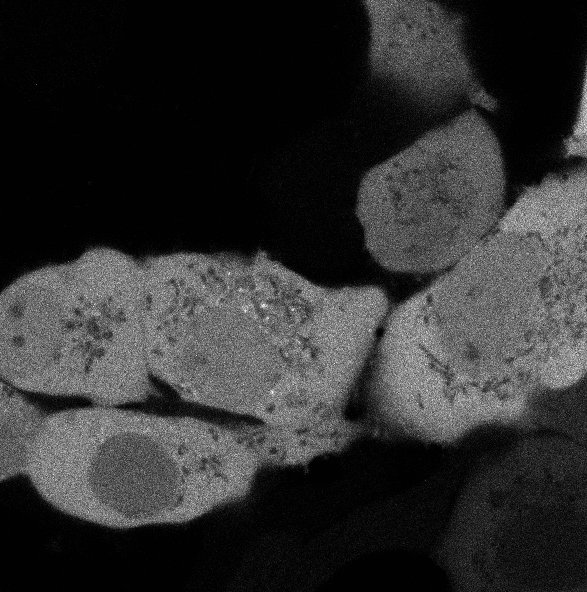

Supplement: Supplementary file 7 — Source data Fig. 6 [file 44318_2024_96_MOESM7_ESM.zip › Figure 6/6D/6D-PITPNB-C94S-control before VT.jpg]

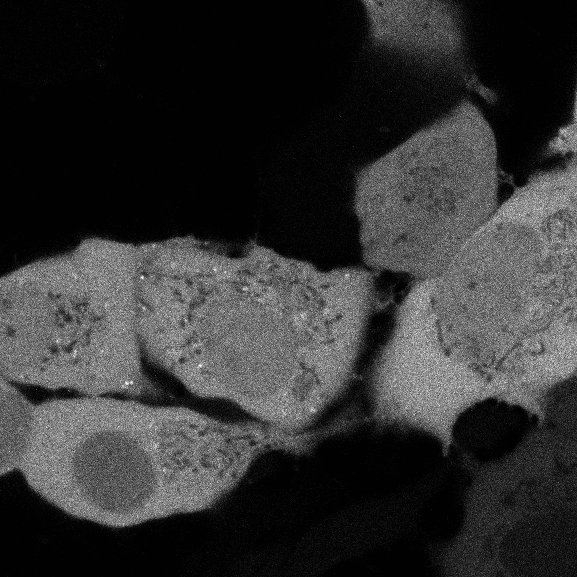

Supplement: Supplementary file 7 — Source data Fig. 6 [file 44318_2024_96_MOESM7_ESM.zip › Figure 6/6D/6D-PITPNB-C94S-VT 10 min.jpg]

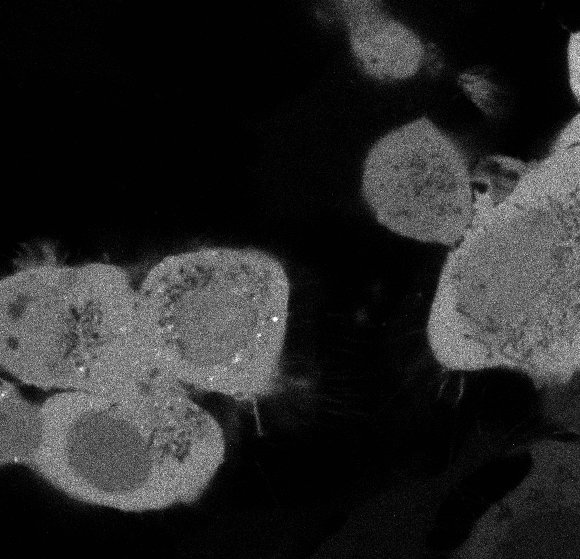

Supplement: Supplementary file 7 — Source data Fig. 6 [file 44318_2024_96_MOESM7_ESM.zip › Figure 6/6D/6D-PITPNB-C94S-VT+AngII 5 min.jpg]
