# Supplementary material for: Non-vesicular phosphatidylinositol transfer plays critical roles in defining organelle lipid composition
Source: EMBO J. 2024 Apr 16;43(10):6. doi: 10.1038/s44318-024-00096-3 (PMC11099152; doi:10.1038/s44318-024-00096-3)
Supplement: Supplementary file 5 — Source data Fig. 4 [file 44318_2024_96_MOESM5_ESM.zip › Figure 4/Fig4-wwPDB X-ray struture val report.pdf]

Full wwPDB X-ray Structure Validation Report (i)

Jul 19, 2023 – 03:09 pm BST

PDB ID	:	8PQO
Title	:	PITP in complex with the inhibitor VT01545
Deposited on	:	2023-07-11
Resolution	:	2.30 Å(reported)

This wwPDB validation report is for manuscript review

This is a Full wwPDB X-ray Structure Validation Report.

This report is produced by the wwPDB biocuration pipeline after annotation of the structure.

We welcome your comments at *validation@mail.wwpdb.org*
A user guide is available at
https://www.wwpdb.org/validation/2017/XrayValidationReportHelp
with specific help available everywhere you see the (i) symbol.

The types of validation reports are described at
http://www.wwpdb.org/validation/2017/FAQs#types.

For Manuscript Review

The following versions of software and data (see references (i)) were used in the production of this report:

MolProbity	:	4.02b-467
Mogul	:	1.8.4, CSD as541be (2020)
Xtriage (Phenix)	:	1.13
EDS	:	2.34
buster-report	:	1.1.7 (2018)
Percentile statistics	:	20191225.v01 (using entries in the PDB archive December 25th 2019)
Refmac	:	5.8.0158
CCP4	:	7.0.044 (Gargrove)
Ideal geometry (proteins)	:	Engh & Huber (2001)
Ideal geometry (DNA, RNA)	:	Parkinson et al. (1996)

1 Overall quality at a glance (i)

The following experimental techniques were used to determine the structure:
X-RAY DIFFRACTION

The reported resolution of this entry is 2.30 Å.

Percentile scores (ranging between 0-100) for global validation metrics of the entry are shown in the following graphic. The table shows the number of entries on which the scores are based.

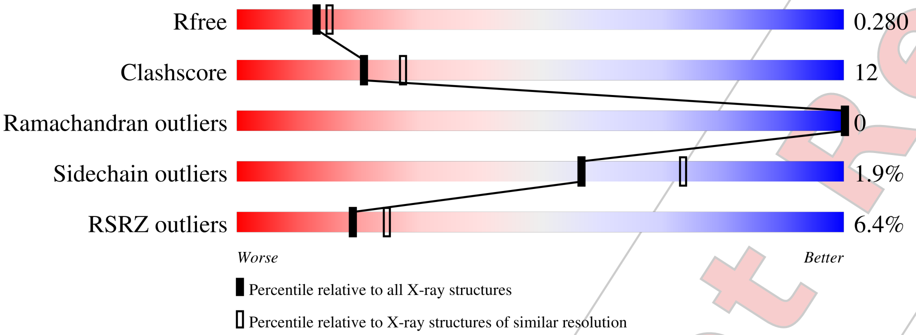

Metric	Whole archive (#Entries)	Similar resolution (#Entries, resolution range(Å))
R_{free}	130704	5042 (2.30-2.30)
Clashscore	141614	5643 (2.30-2.30)
Ramachandran outliers	138981	5575 (2.30-2.30)
Sidechain outliers	138945	5575 (2.30-2.30)
RSRZ outliers	127900	4938 (2.30-2.30)

The table below summarises the geometric issues observed across the polymeric chains and their fit to the electron density. The red, orange, yellow and green segments of the lower bar indicate the fraction of residues that contain outliers for >=3, 2, 1 and 0 types of geometric quality criteria respectively. A grey segment represents the fraction of residues that are not modelled. The numeric value for each fraction is indicated below the corresponding segment, with a dot representing fractions <=5% The upper red bar (where present) indicates the fraction of residues that have poor fit to the electron density. The numeric value is given above the bar.

Mol	Chain	Length	Quality of chain
1	C	270	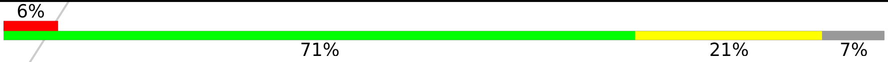

The following table lists non-polymeric compounds, carbohydrate monomers and non-standard residues in protein, DNA, RNA chains that are outliers for geometric or electron-density-fit criteria:

For Manuscript Review

Mol	Type	Chain	Res	Chirality	Geometry	Clashes	Electron density
3	GOL	C	303	-	-	-	X

For Manuscript Review

2 Entry composition (i)

There are 11 unique types of molecules in this entry. The entry contains 2411 atoms, of which 29 are hydrogens and 0 are deuteriums.

In the tables below, the ZeroOcc column contains the number of atoms modelled with zero occupancy, the AltConf column contains the number of residues with at least one atom in alternate conformation and the Trace column contains the number of residues modelled with at most 2 atoms.

- Molecule 1 is a protein called Phosphatidylinositol transfer protein alpha isoform.

Mol	Chain	Residues	Atoms					ZeroOcc	AltConf	Trace
			Total	C	N	O	S			
1	C	251	2066	1326	352	380	8	0	1	0

There is a discrepancy between the modelled and reference sequences:

Chain	Residue	Modelled	Actual	Comment	Reference
C	0	SER	-	expression tag	UNP Q00169

- Molecule 2 is IMIDAZOLE (three-letter code: IMD) (formula: $C_3H_5N_2$).

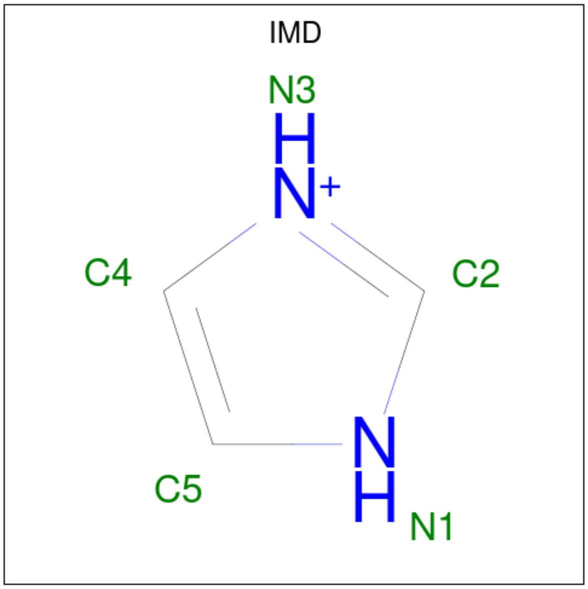

Mol	Chain	Residues	Atoms				ZeroOcc	AltConf
			Total	C	H	N		
2	C	1	10	3	5	2	0	0

- Molecule 3 is GLYCEROL (three-letter code: GOL) (formula: $C_3H_8O_3$).

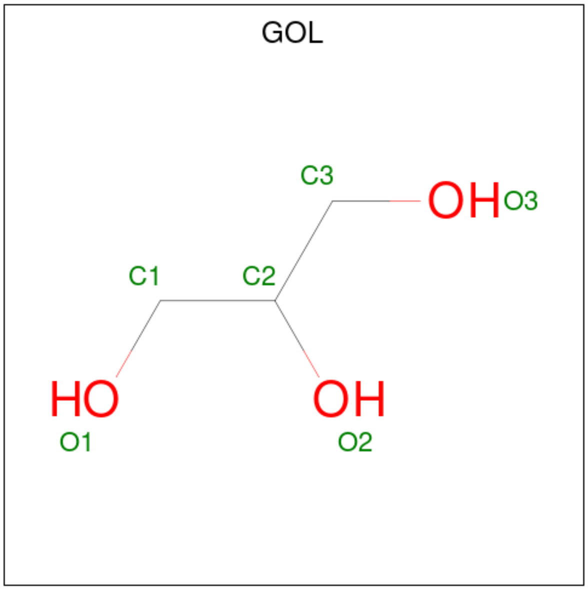

Mol	Chain	Residues	Atoms	ZeroOcc	AltConf
3	C	1	Total C H O<br>14 3 8 3	0	0
3	C	1	Total C H O<br>14 3 8 3	0	0
3	C	1	Total C H O<br>14 3 8 3	0	0

- Molecule 4 is NICKEL (II) ION (three-letter code: NI) (formula: Ni).

Mol	Chain	Residues	Atoms	ZeroOcc	AltConf
4	C	1	Total Ni<br>1 1	0	0

- Molecule 5 is (2 {R},4 {R})-2,4-dimethyloctanal (three-letter code: F8O) (formula: $C_{10}H_{20}O$) (labeled as "Ligand of Interest" by depositor).

For Manuscript Review

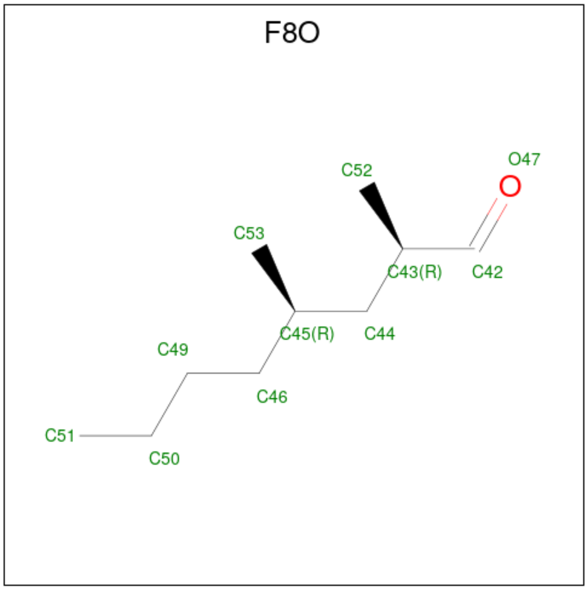

Mol	Chain	Residues	Atoms	ZeroOcc	AltConf
5	C	1	Total C O<br>11 10 1	0	0

- Molecule 6 is (2 {R})-4-methyl-2-(methylamino)pentanamide (three-letter code: FDO) (formula: $C_7H_{16}N_2O$) (labeled as "Ligand of Interest" by depositor).

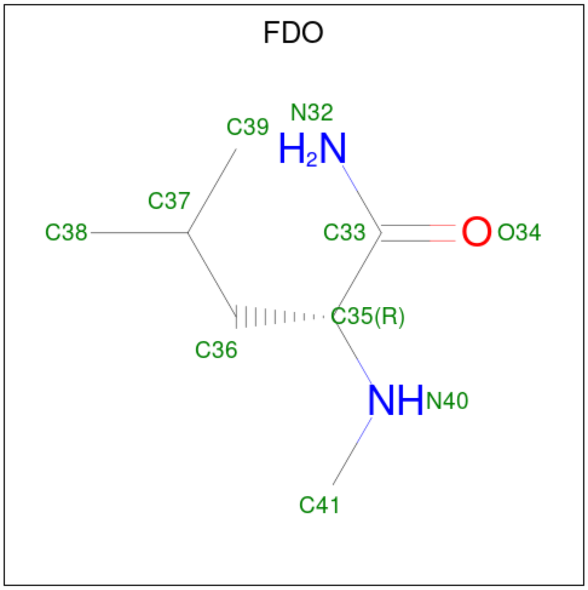

Mol	Chain	Residues	Atoms	ZeroOcc	AltConf
6	C	1	Total C N O<br>10 7 2 1	0	0

- Molecule 7 is (3R)-butane-1,3-diol (three-letter code: BU4) (formula: $C_4H_{10}O_2$) (labeled as "Ligand of Interest" by depositor).

For Manuscript Review

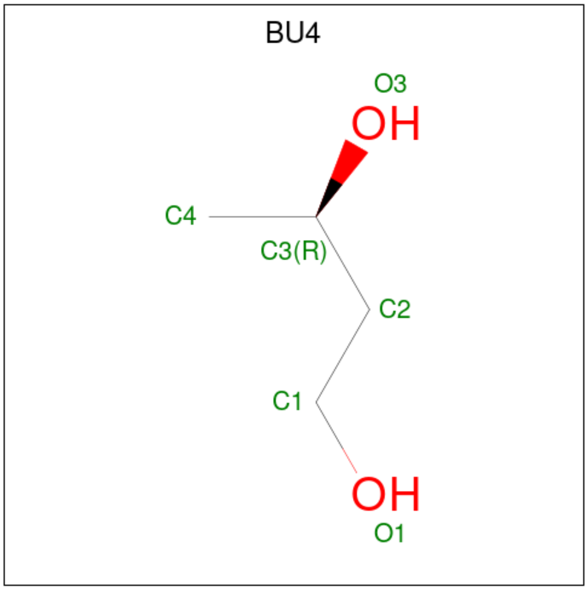

Mol	Chain	Residues	Atoms			ZeroOcc	AltConf
			Total	C	O		
7	C	1	6	4	2	0	0

- Molecule 8 is (2 {R})-3-methyl-2-(methylamino)butanoic acid (three-letter code: MV9) (formula: $C_6H_{13}NO_2$) (labeled as "Ligand of Interest" by depositor).

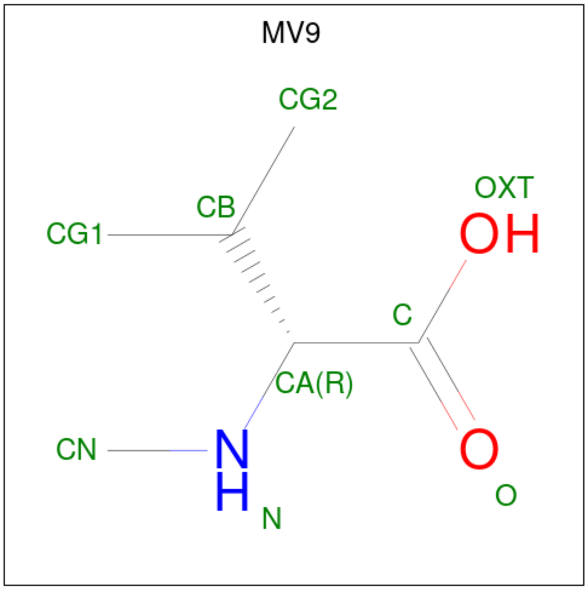

Mol	Chain	Residues	Atoms				ZeroOcc	AltConf
			Total	C	N	O		
8	C	1	8	6	1	1	0	0

- Molecule 9 is (4R)-4-hydroxy-D-proline (three-letter code: UYA) (formula: $C_5H_9NO_3$) (labeled as "Ligand of Interest" by depositor).

For Manuscript Review

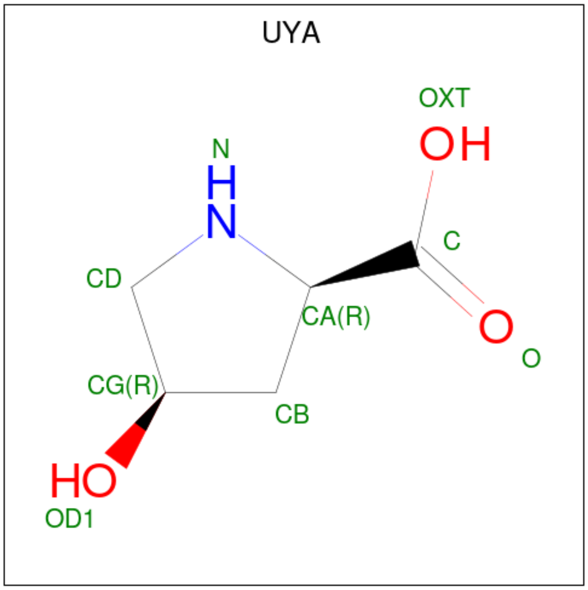

Mol	Chain	Residues	Atoms	ZeroOcc	AltConf
9	C	1	Total C N O<br>8 5 1 2	0	0

- Molecule 10 is (2 {R})-2-azanyl-3-[(2 {S},3 {R})-2-methyl-5-oxidanylidene-pyrrolidin-3-y l]sulfanyl-propanal (three-letter code: FH6) (formula: $C_8H_{14}N_2O_2S$) (labeled as "Ligand of Interest" by depositor).

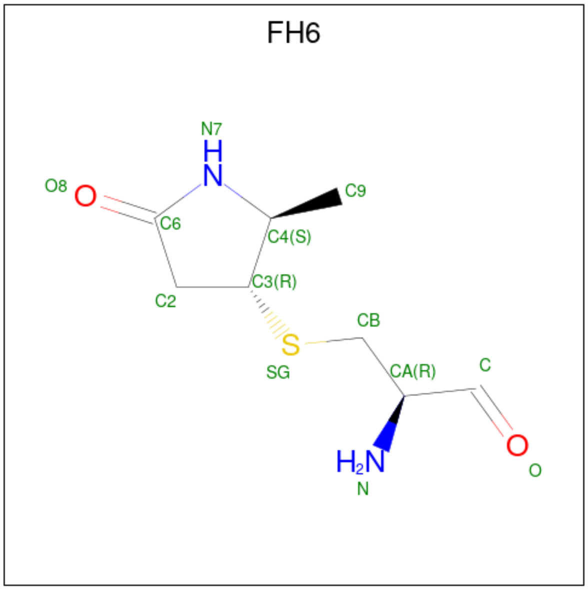

Mol	Chain	Residues	Atoms	ZeroOcc	AltConf
10	C	1	Total C N O S<br>13 8 2 2 1	0	0

- Molecule 11 is water.

For Manuscript Review

Mol	Chain	Residues	Atoms		ZeroOcc	AltConf
			Total	O		
11	C	236	236	236	0	0

For Manuscript Review

3 Residue-property plots (i)

These plots are drawn for all protein, RNA, DNA and oligosaccharide chains in the entry. The first graphic for a chain summarises the proportions of the various outlier classes displayed in the second graphic. The second graphic shows the sequence view annotated by issues in geometry and electron density. Residues are color-coded according to the number of geometric quality criteria for which they contain at least one outlier: green = 0, yellow = 1, orange = 2 and red = 3 or more. A red dot above a residue indicates a poor fit to the electron density (RSRZ > 2). Stretches of 2 or more consecutive residues without any outlier are shown as a green connector. Residues present in the sample, but not in the model, are shown in grey.

- Molecule 1: Phosphatidylinositol transfer protein alpha isoform

Chain C:

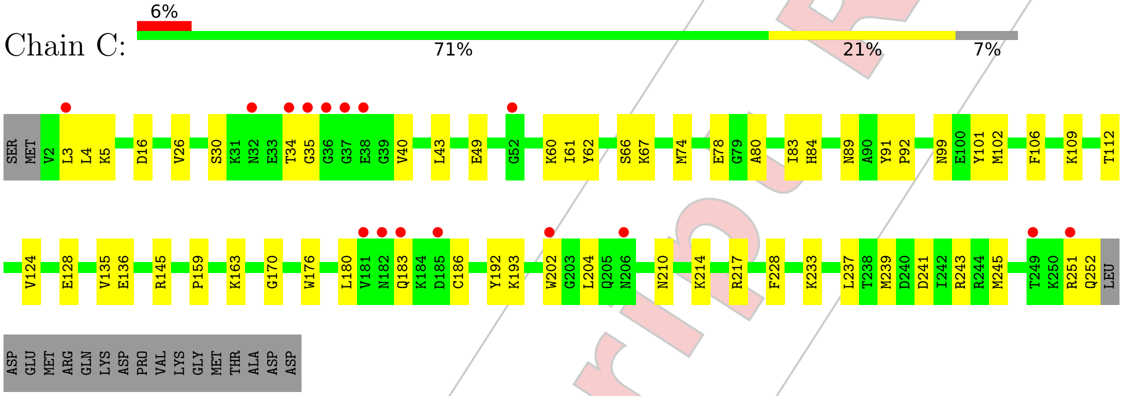

For Manuscript Review

4 Data and refinement statistics (i)

Property	Value	Source
Space group	P 2 21 21	Depositor
Cell constants<br>a, b, c, α, β, γ	50.33Å 81.91Å 94.70Å<br>90.00° 90.00° 90.00°	Depositor
Resolution (Å)	25.42 – 2.30<br>25.42 – 2.30	Depositor<br>EDS
% Data completeness<br>(in resolution range)	95.7 (25.42-2.30)<br>95.6 (25.42-2.30)	Depositor<br>EDS
R_{merge}	0.13	Depositor
R_{sym}	(Not available)	Depositor
$< I/\sigma(I) >$ [1]	3.45 (at 2.31Å)	Xtriage
Refinement program	PHENIX (1.20.1_4487: ???)	Depositor
R, R_{free}	0.235 , 0.282<br>0.235 , 0.280	Depositor<br>DCC
R_{free} test set	824 reflections (4.79%)	wwPDB-VP
Wilson B-factor (Å^2)	33.3	Xtriage
Anisotropy	0.623	Xtriage
Bulk solvent k_{sol}(e/Å^3), B_{sol}(Å^2)	0.32 , 44.3	EDS
L-test for twinning[2]	$< \|L\| > = 0.50$, $< L^2 > = 0.34$	Xtriage
Estimated twinning fraction	No twinning to report.	Xtriage
F_o,F_c correlation	0.92	EDS
Total number of atoms	2411	wwPDB-VP
Average B, all atoms (Å^2)	38.0	wwPDB-VP

Xtriage's analysis on translational NCS is as follows: *The largest off-origin peak in the Patterson function is 6.58% of the height of the origin peak. No significant pseudotranslation is detected.*

[1] Intensities estimated from amplitudes.

[2] Theoretical values of $< |L| >$, $< L^2 >$ for acentric reflections are 0.5, 0.333 respectively for untwinned datasets, and 0.375, 0.2 for perfectly twinned datasets.

For Manuscript Review

5 Model quality (i)

5.1 Standard geometry (i)

Bond lengths and bond angles in the following residue types are not validated in this section: UYA, NI, IMD, GOL, BU4, FDO, F8O, MV9, FH6

The Z score for a bond length (or angle) is the number of standard deviations the observed value is removed from the expected value. A bond length (or angle) with $|Z| > 5$ is considered an outlier worth inspection. RMSZ is the root-mean-square of all Z scores of the bond lengths (or angles).

Mol	Chain	Bond lengths		Bond angles	
		RMSZ	$\#\|Z\| > 5$	RMSZ	$\#\|Z\| > 5$
1	C	0.29	0/2120	0.51	0/2874

There are no bond length outliers.

There are no bond angle outliers.

There are no chirality outliers.

There are no planarity outliers.

5.2 Too-close contacts (i)

In the following table, the Non-H and H(model) columns list the number of non-hydrogen atoms and hydrogen atoms in the chain respectively. The H(added) column lists the number of hydrogen atoms added and optimized by MolProbity. The Clashes column lists the number of clashes within the asymmetric unit, whereas Symm-Clashes lists symmetry-related clashes.

Mol	Chain	Non-H	H(model)	H(added)	Clashes	Symm-Clashes
1	C	2066	0	1987	49	1
2	C	5	5	4	1	0
3	C	18	24	24	3	1
4	C	1	0	0	0	0
5	C	11	0	0	0	0
6	C	10	0	0	0	0
7	C	6	0	6	0	0
8	C	8	0	0	0	0
9	C	8	0	0	2	0
10	C	13	0	0	0	0
11	C	236	0	0	19	1
All	All	2382	29	2021	51	2

The all-atom clashscore is defined as the number of clashes found per 1000 atoms (including

For Manuscript Review

hydrogen atoms). The all-atom clashscore for this structure is 12.

All (51) close contacts within the same asymmetric unit are listed below, sorted by their clash magnitude.

Atom-1	Atom-2	Interatomic distance (Å)	Clash overlap (Å)
1:C:136:GLU:OE2	11:C:401:HOH:O	1.81	0.98
1:C:78:GLU:OE1	11:C:403:HOH:O	1.90	0.89
1:C:89:ASN:HB2	11:C:413:HOH:O	1.71	0.89
1:C:102:MET:HB3	11:C:439:HOH:O	1.73	0.87
1:C:102:MET:HG2	1:C:106:PHE:HB2	1.56	0.85
1:C:109:LYS:NZ	11:C:402:HOH:O	1.84	0.84
1:C:35:GLY:O	1:C:67:LYS:NZ	2.15	0.79
1:C:183:GLN:O	11:C:404:HOH:O	2.05	0.73
1:C:16:ASP:OD2	11:C:405:HOH:O	2.06	0.72
1:C:186:CYS:N	11:C:411:HOH:O	2.20	0.72
1:C:202:TRP:HB2	1:C:204:LEU:HD12	1.73	0.69
1:C:210:ASN:OD1	11:C:406:HOH:O	2.10	0.69
1:C:128:GLU:HA	1:C:128:GLU:OE1	1.93	0.69
1:C:60:LYS:NZ	3:C:305:GOL:O2	2.25	0.66
1:C:145[B]:ARG:HG3	1:C:145[B]:ARG:HH11	1.61	0.66
1:C:102:MET:HG2	1:C:106:PHE:CB	2.27	0.65
1:C:163:LYS:HG3	1:C:170:GLY:O	1.99	0.63
1:C:145[A]:ARG:NH2	11:C:408:HOH:O	2.16	0.63
1:C:186:CYS:CA	11:C:411:HOH:O	2.46	0.63
1:C:239:MET:O	1:C:243:ARG:HG3	2.02	0.58
1:C:3:LEU:HD21	1:C:5:LYS:NZ	2.19	0.57
1:C:124:VAL:O	11:C:409:HOH:O	2.17	0.57
1:C:241:ASP:O	1:C:245:MET:HG3	2.05	0.57
1:C:40:VAL:HG22	1:C:62:TYR:CD2	2.40	0.56
1:C:252:GLN:N	1:C:252:GLN:OE1	2.39	0.55
1:C:26:VAL:HB	11:C:443:HOH:O	2.06	0.54
1:C:233:LYS:NZ	11:C:415:HOH:O	2.31	0.53
1:C:4:LEU:HB3	1:C:135:VAL:HG22	1.92	0.52
1:C:145[B]:ARG:HG3	1:C:145[B]:ARG:NH1	2.24	0.52
1:C:40:VAL:HG22	1:C:62:TYR:CE2	2.45	0.51
1:C:214:LYS:HD3	11:C:444:HOH:O	2.10	0.51
1:C:217:ARG:HG2	11:C:574:HOH:O	2.12	0.50
1:C:49:GLU:HG2	11:C:558:HOH:O	2.12	0.50
1:C:80:ALA:O	1:C:99:ASN:ND2	2.46	0.47
1:C:112:THR:HA	1:C:192:TYR:O	2.14	0.47
3:C:305:GOL:H12	11:C:455:HOH:O	2.14	0.47
1:C:251:ARG:HB2	1:C:252:GLN:OE1	2.15	0.47
1:C:159:PRO:HB3	1:C:228:PHE:CD2	2.49	0.47

Continued on next page...

For Manuscript Review

Continued from previous page...

Atom-1	Atom-2	Interatomic distance (Å)	Clash overlap (Å)
1:C:83:ILE:N	1:C:83:ILE:HD12	2.31	0.45
1:C:193:LYS:HZ1	9:C:310:UYA:CA	2.30	0.44
1:C:193:LYS:HZ1	9:C:310:UYA:CB	2.31	0.44
1:C:241:ASP:O	1:C:245:MET:CG	2.66	0.43
1:C:78:GLU:O	1:C:101:TYR:HB2	2.19	0.43
1:C:61:ILE:HD12	2:C:301:IMD:C4	2.50	0.42
3:C:305:GOL:H32	11:C:414:HOH:O	2.19	0.42
1:C:3:LEU:HD21	1:C:5:LYS:HZ1	1.83	0.42
1:C:176:TRP:CZ3	1:C:180:LEU:HD11	2.55	0.42
1:C:237:LEU:HD13	1:C:245:MET:CE	2.51	0.41
1:C:61:ILE:HD11	1:C:84:HIS:CE1	2.56	0.40
1:C:61:ILE:CD1	1:C:84:HIS:CE1	3.05	0.40
1:C:91:TYR:CG	1:C:92:PRO:HA	2.57	0.40

All (2) symmetry-related close contacts are listed below. The label for Atom-2 includes the symmetry operator and encoded unit-cell translations to be applied.

Atom-1	Atom-2	Interatomic distance (Å)	Clash overlap (Å)
1:C:43:LEU:O	3:C:303:GOL:O2[4_654]	2.00	0.20
11:C:410:HOH:O	11:C:593:HOH:O[2_565]	2.17	0.03

5.3 Torsion angles (i)

5.3.1 Protein backbone (i)

In the following table, the Percentiles column shows the percent Ramachandran outliers of the chain as a percentile score with respect to all X-ray entries followed by that with respect to entries of similar resolution.

The Analysed column shows the number of residues for which the backbone conformation was analysed, and the total number of residues.

Mol	Chain	Analysed	Favoured	Allowed	Outliers	Percentiles	
1	C	248/270 (92%)	244 (98%)	4 (2%)	0	100	100

There are no Ramachandran outliers to report.

For Manuscript Review

5.3.2 Protein sidechains (i)

In the following table, the Percentiles column shows the percent sidechain outliers of the chain as a percentile score with respect to all X-ray entries followed by that with respect to entries of similar resolution.

The Analysed column shows the number of residues for which the sidechain conformation was analysed, and the total number of residues.

Mol	Chain	Analysed	Rotameric	Outliers	Percentiles
1	C	217/243 (89%)	213 (98%)	4 (2%)	59 75

All (4) residues with a non-rotameric sidechain are listed below:

Mol	Chain	Res	Type
1	C	30	SER
1	C	34	THR
1	C	66	SER
1	C	74	MET

Sometimes sidechains can be flipped to improve hydrogen bonding and reduce clashes. There are no such sidechains identified.

5.3.3 RNA (i)

There are no RNA molecules in this entry.

5.4 Non-standard residues in protein, DNA, RNA chains (i)

There are no non-standard protein/DNA/RNA residues in this entry.

5.5 Carbohydrates (i)

There are no monosaccharides in this entry.

5.6 Ligand geometry (i)

Of 11 ligands modelled in this entry, 1 is monoatomic - leaving 10 for Mogul analysis.

In the following table, the Counts columns list the number of bonds (or angles) for which Mogul statistics could be retrieved, the number of bonds (or angles) that are observed in the model and the number of bonds (or angles) that are defined in the Chemical Component Dictionary. The

For Manuscript Review

Link column lists molecule types, if any, to which the group is linked. The Z score for a bond length (or angle) is the number of standard deviations the observed value is removed from the expected value. A bond length (or angle) with $|Z| > 2$ is considered an outlier worth inspection. RMSZ is the root-mean-square of all Z scores of the bond lengths (or angles).

Mol	Type	Chain	Res	Link	Bond lengths			Bond angles		
					Counts	RMSZ	$\#\|Z\| > 2$	Counts	RMSZ	$\#\|Z\| > 2$
3	GOL	C	303	-	5,5,5	0.12	0	5,5,5	0.44	0
8	MV9	C	309	9,7	6,7,8	0.76	0	7,8,10	1.71	2 (28%)
7	BU4	C	308	6,8	5,5,5	1.92	1 (20%)	5,5,5	1.32	1 (20%)
6	FDO	C	307	7,5	9,9,9	0.52	0	10,11,11	1.32	1 (10%)
5	F8O	C	306	6	10,10,10	0.49	0	10,11,11	1.14	1 (10%)
2	IMD	C	301	4	3,5,5	0.38	0	4,5,5	0.58	0
3	GOL	C	302	-	5,5,5	0.99	0	5,5,5	1.24	1 (20%)
3	GOL	C	305	-	5,5,5	0.09	0	5,5,5	0.23	0
9	UYA	C	310	10,8	7,8,9	1.99	1 (14%)	7,10,12	1.60	2 (28%)
10	FH6	C	311	9,1	11,13,13	1.54	2 (18%)	6,17,17	1.12	1 (16%)

In the following table, the Chirals column lists the number of chiral outliers, the number of chiral centers analysed, the number of these observed in the model and the number defined in the Chemical Component Dictionary. Similar counts are reported in the Torsion and Rings columns. '-' means no outliers of that kind were identified.

Mol	Type	Chain	Res	Link	Chirals	Torsions	Rings
3	GOL	C	303	-	-	0/4/4/4	-
8	MV9	C	309	9,7	-	1/6/8/10	-
7	BU4	C	308	6,8	-	0/3/3/3	-
6	FDO	C	307	7,5	-	6/10/10/10	-
5	F8O	C	306	6	-	4/9/10/10	-
2	IMD	C	301	4	-	-	0/1/1/1
3	GOL	C	302	-	-	4/4/4/4	-
3	GOL	C	305	-	-	2/4/4/4	-
9	UYA	C	310	10,8	-	0/2/11/13	0/1/1/1
10	FH6	C	311	9,1	-	0/5/19/19	0/1/1/1

All (4) bond length outliers are listed below:

Mol	Chain	Res	Type	Atoms	Z	Observed(Å)	Ideal(Å)
9	C	310	UYA	OXT-C	-4.67	1.22	1.42
10	C	311	FH6	CB-SG	-4.15	1.77	1.82
7	C	308	BU4	O1-C1	-3.75	1.22	1.42
10	C	311	FH6	C6-N7	2.22	1.40	1.34

For Manuscript Review

All (9) bond angle outliers are listed below:

Mol	Chain	Res	Type	Atoms	Z	Observed(°)	Ideal(°)
5	C	306	F8O	O47-C42-C43	-3.14	118.15	125.32
6	C	307	FDO	C36-C35-C33	3.10	115.95	109.44
7	C	308	BU4	C1-C2-C3	2.77	121.66	114.04
8	C	309	MV9	CB-CA-N	2.71	114.70	111.17
8	C	309	MV9	O-C-CA	-2.68	117.35	124.83
9	C	310	UYA	OXT-C-CA	2.64	118.78	111.95
3	C	302	GOL	C3-C2-C1	-2.47	102.11	111.70
10	C	311	FH6	CA-CB-SG	2.14	121.22	113.58
9	C	310	UYA	CB-CG-CD	2.04	105.77	103.27

There are no chirality outliers.

All (17) torsion outliers are listed below:

Mol	Chain	Res	Type	Atoms
3	C	302	GOL	O1-C1-C2-O2
3	C	302	GOL	O1-C1-C2-C3
5	C	306	F8O	O47-C42-C43-C44
5	C	306	F8O	C43-C44-C45-C53
6	C	307	FDO	N32-C33-C35-C36
6	C	307	FDO	O34-C33-C35-C36
5	C	306	F8O	C45-C46-C49-C50
3	C	302	GOL	O2-C2-C3-O3
3	C	302	GOL	C1-C2-C3-O3
3	C	305	GOL	C1-C2-C3-O3
6	C	307	FDO	C35-C36-C37-C39
5	C	306	F8O	C46-C49-C50-C51
6	C	307	FDO	C35-C36-C37-C38
3	C	305	GOL	O2-C2-C3-O3
6	C	307	FDO	C33-C35-N40-C41
8	C	309	MV9	CB-CA-N-CN
6	C	307	FDO	C36-C35-N40-C41

There are no ring outliers.

4 monomers are involved in 7 short contacts:

Mol	Chain	Res	Type	Clashes	Symm-Clashes
3	C	303	GOL	0	1
2	C	301	IMD	1	0
3	C	305	GOL	3	0
9	C	310	UYA	2	0

For Manuscript Review

The following is a two-dimensional graphical depiction of Mogul quality analysis of bond lengths, bond angles, torsion angles, and ring geometry for all instances of the Ligand of Interest. In addition, ligands with molecular weight > 250 and outliers as shown on the validation Tables will also be included. For torsion angles, if less then 5% of the Mogul distribution of torsion angles is within 10 degrees of the torsion angle in question, then that torsion angle is considered an outlier. Any bond that is central to one or more torsion angles identified as an outlier by Mogul will be highlighted in the graph. For rings, the root-mean-square deviation (RMSD) between the ring in question and similar rings identified by Mogul is calculated over all ring torsion angles. If the average RMSD is greater than 60 degrees and the minimal RMSD between the ring in question and any Mogul-identified rings is also greater than 60 degrees, then that ring is considered an outlier. The outliers are highlighted in purple. The color gray indicates Mogul did not find sufficient equivalents in the CSD to analyse the geometry.

Ligand MV9 C 309

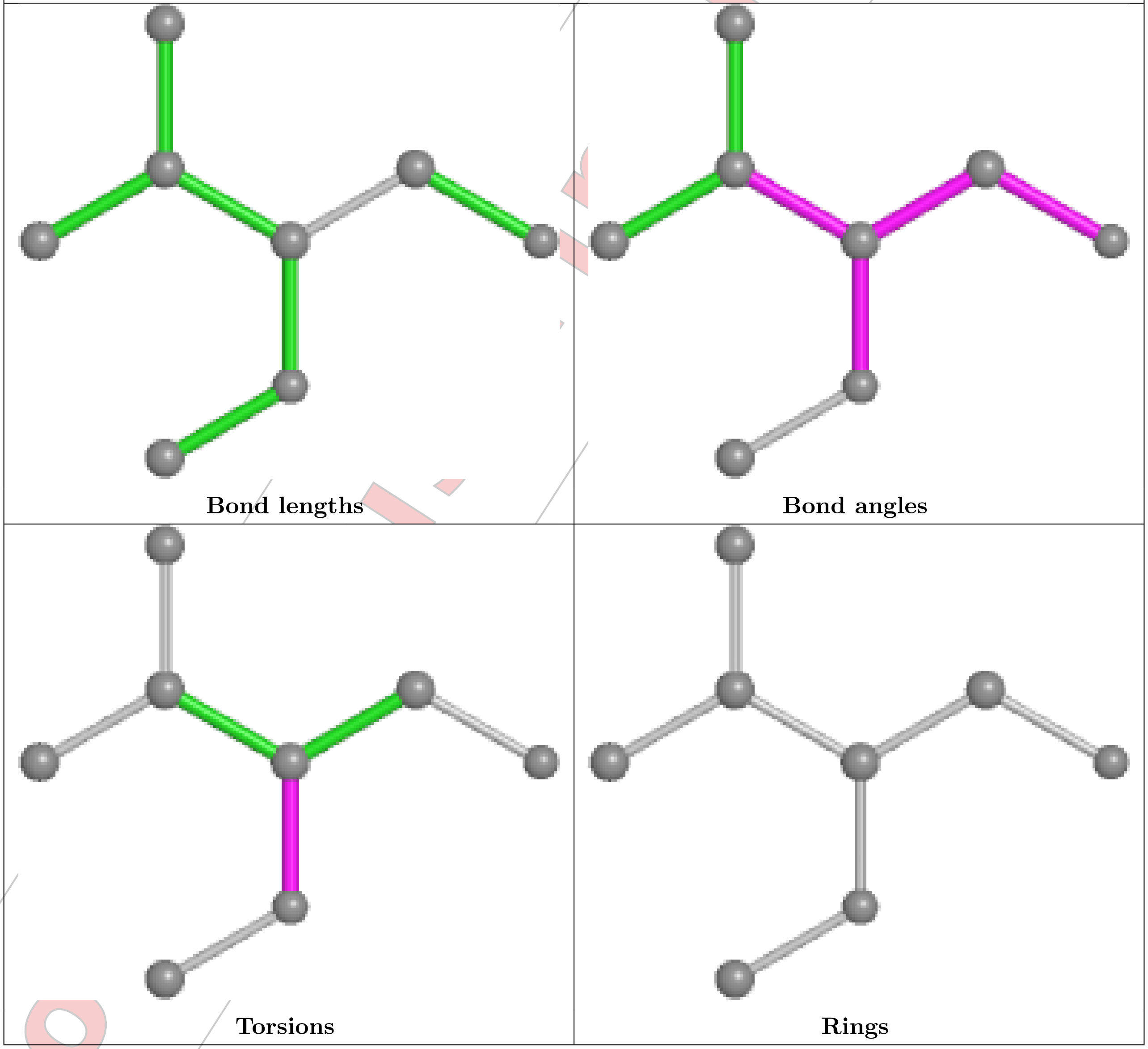

Ligand BU4 C 308

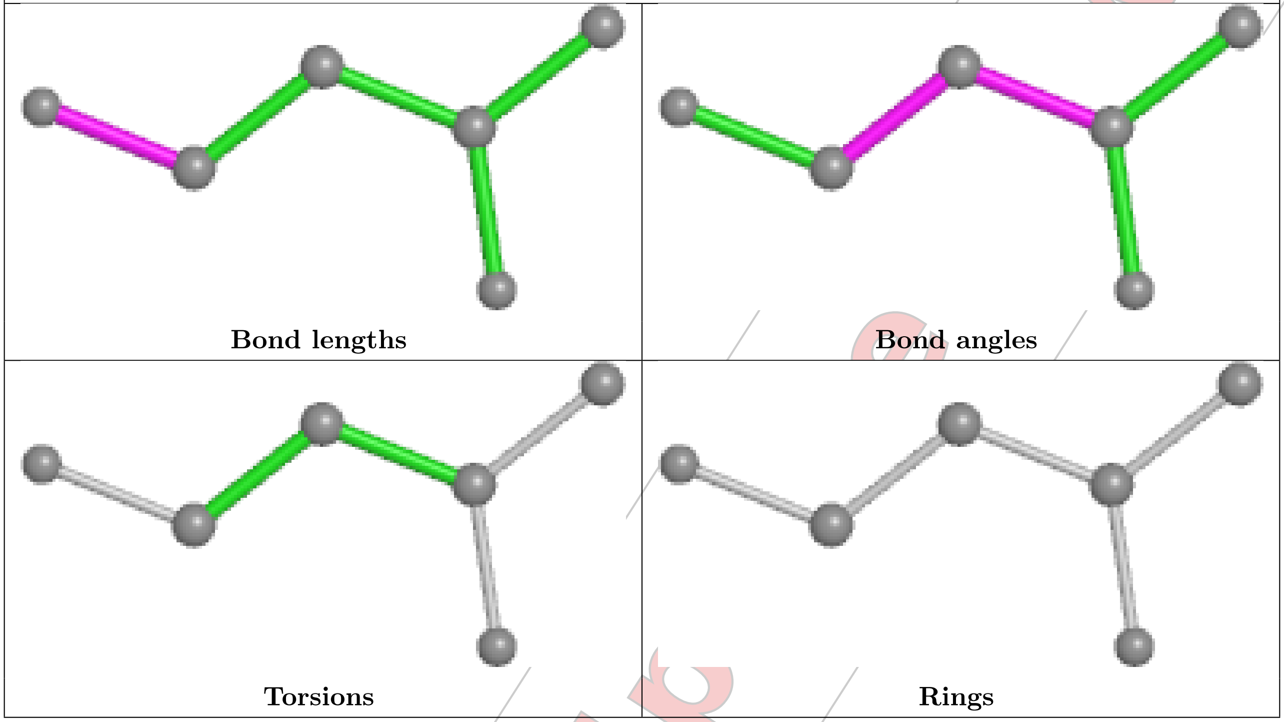

For Manuscript Review

Ligand FDO C 307

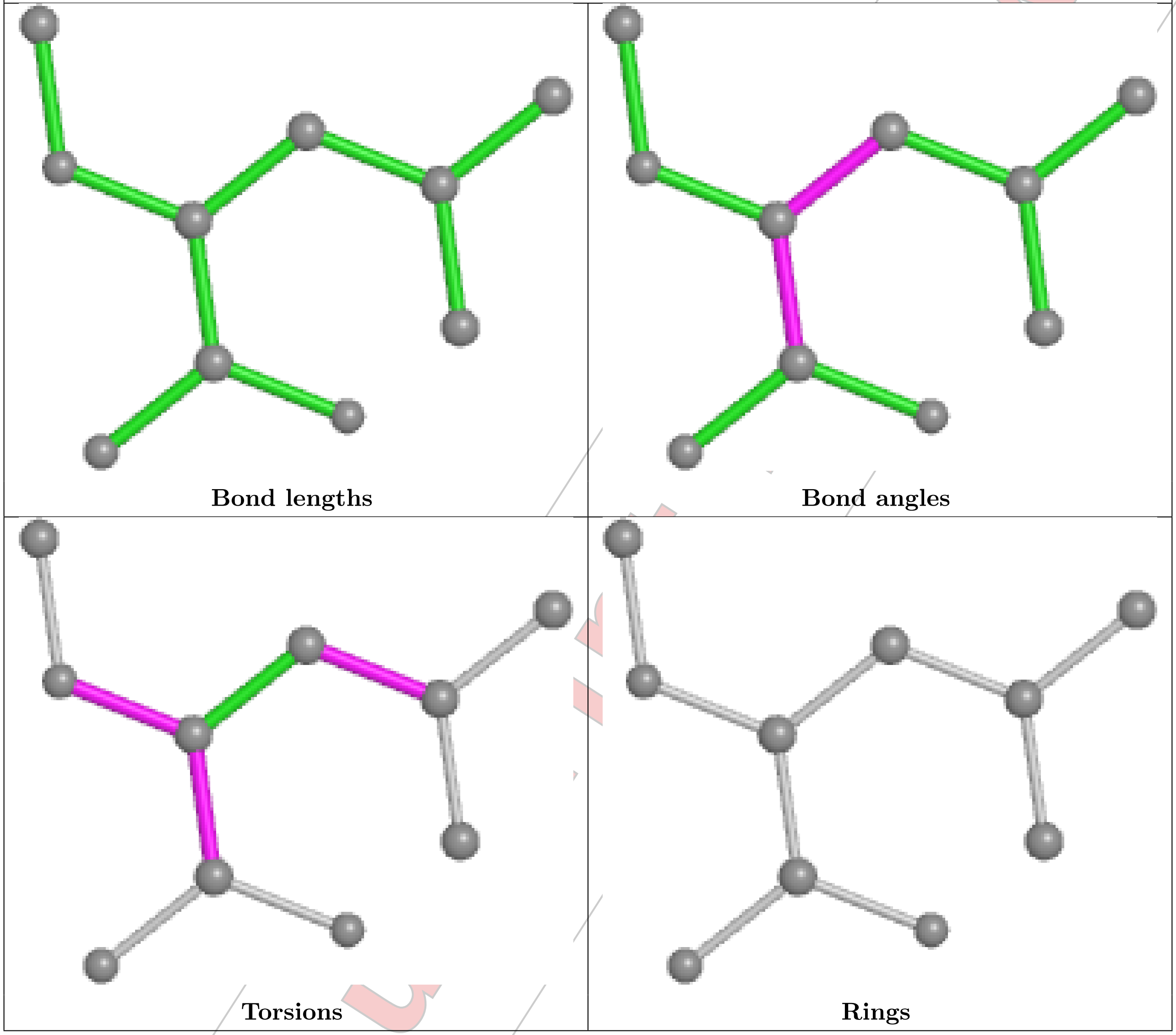

For Manuscript Review

Ligand F8O C 306

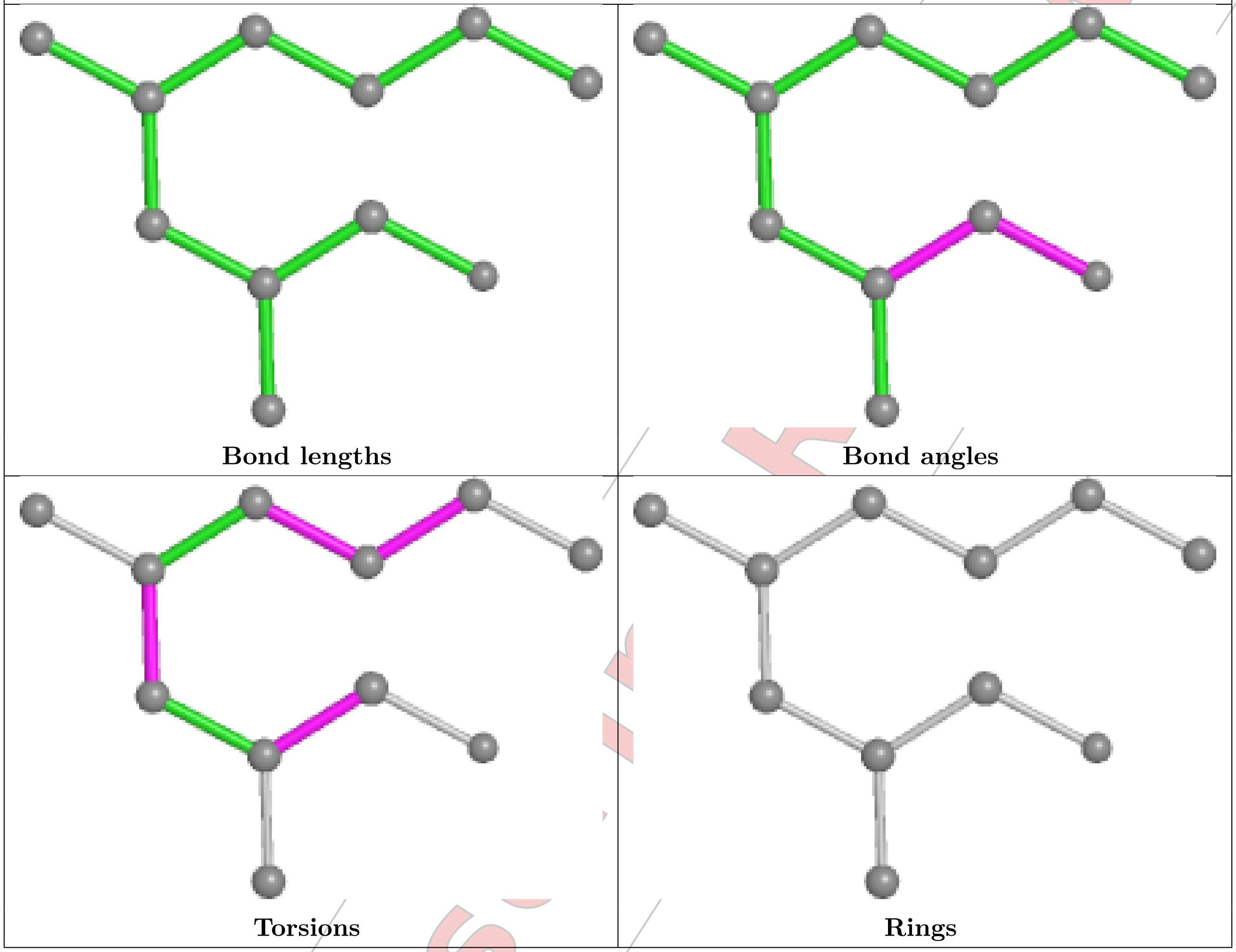

Ligand UYA C 310

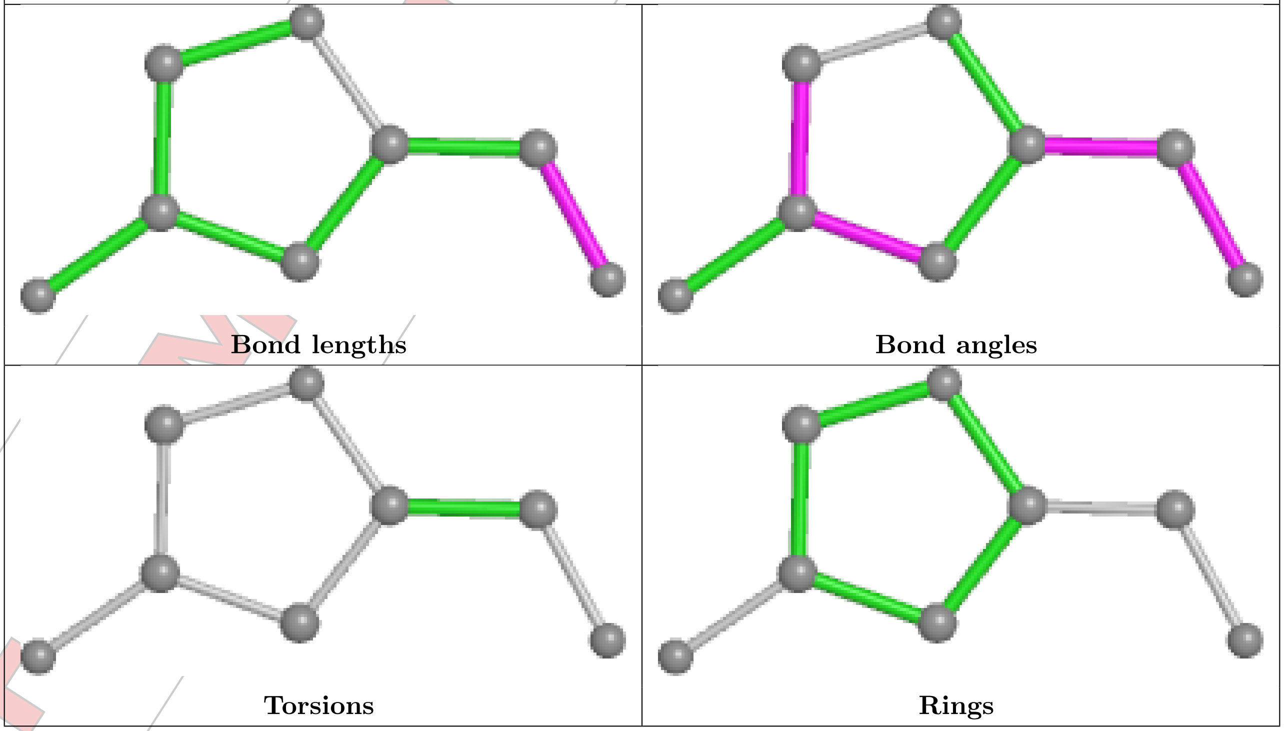

Ligand FH6 C 311

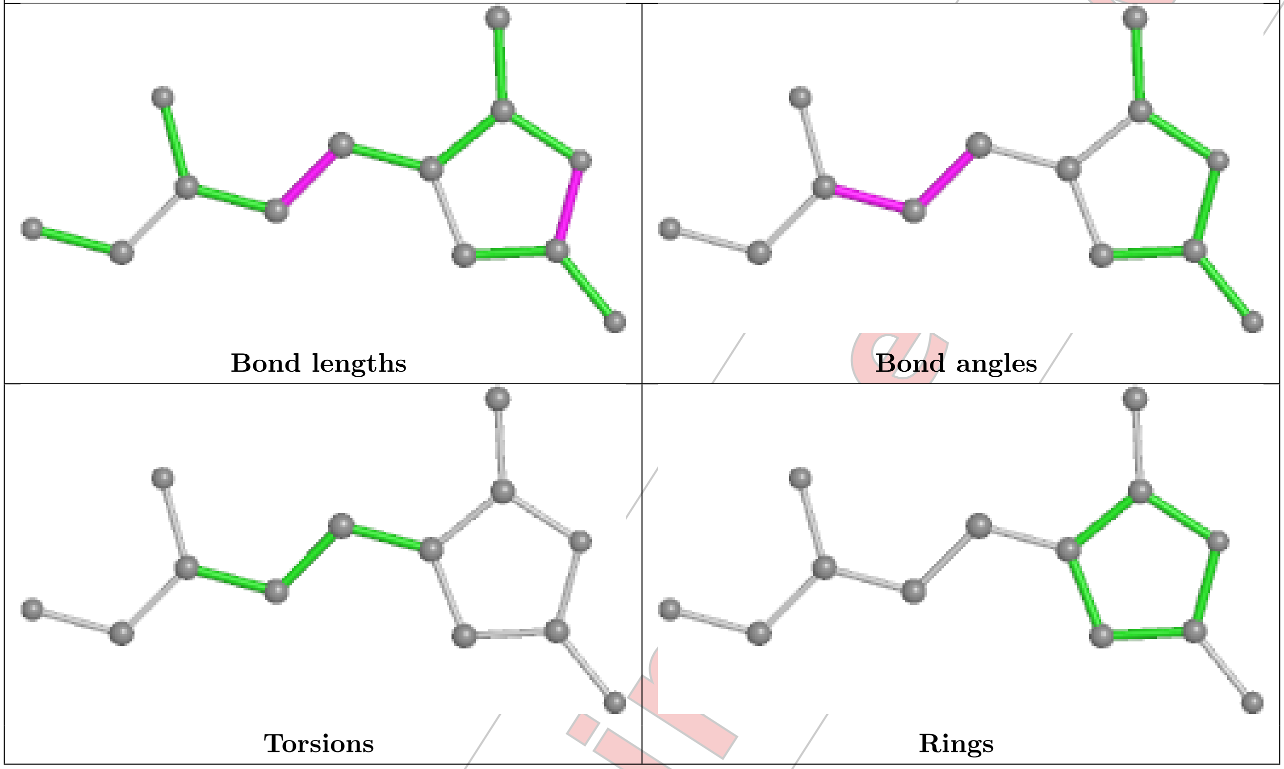

5.7 Other polymers (i)

There are no such residues in this entry.

5.8 Polymer linkage issues (i)

The following chains have linkage breaks:

Mol	Chain	Number of breaks
1	C	1

All chain breaks are listed below:

Model	Chain	Residue-1	Atom-1	Residue-2	Atom-2	Distance (Å)
1	C	93:TYR	C	94:ARG	N	4.64

For Manuscript Review

6 Fit of model and data (i)

6.1 Protein, DNA and RNA chains (i)

In the following table, the column labelled '#RSRZ> 2' contains the number (and percentage) of RSRZ outliers, followed by percent RSRZ outliers for the chain as percentile scores relative to all X-ray entries and entries of similar resolution. The OWAB column contains the minimum, median, 95th percentile and maximum values of the occupancy-weighted average B-factor per residue. The column labelled 'Q< 0.9' lists the number of (and percentage) of residues with an average occupancy less than 0.9.

Mol	Chain	Analysed	<RSRZ>	#RSRZ>2			OWAB(Å^2)	Q<0.9
1	C	251/270 (92%)	0.31	16 (6%)	19	25	17, 33, 74, 95	0

All (16) RSRZ outliers are listed below:

Mol	Chain	Res	Type	RSRZ
1	C	37	GLY	5.0
1	C	35	GLY	4.4
1	C	36	GLY	4.3
1	C	185	ASP	3.8
1	C	32	ASN	3.7
1	C	34	THR	3.4
1	C	251	ARG	3.4
1	C	3	LEU	3.2
1	C	182	ASN	3.1
1	C	52	GLY	3.0
1	C	38	GLU	3.0
1	C	249	THR	2.7
1	C	202	TRP	2.4
1	C	181	VAL	2.4
1	C	183	GLN	2.2
1	C	206	ASN	2.2

6.2 Non-standard residues in protein, DNA, RNA chains (i)

There are no non-standard protein/DNA/RNA residues in this entry.

6.3 Carbohydrates (i)

There are no monosaccharides in this entry.

For Manuscript Review

6.4 Ligands (i)

In the following table, the Atoms column lists the number of modelled atoms in the group and the number defined in the chemical component dictionary. The B-factors column lists the minimum, median, 95th percentile and maximum values of B factors of atoms in the group. The column labelled 'Q< 0.9' lists the number of atoms with occupancy less than 0.9.

Mol	Type	Chain	Res	Atoms	RSCC	RSR	B-factors($Å^2$)	Q<0.9
3	GOL	C	302	6/6	0.73	0.15	56,67,80,80	0
3	GOL	C	303	6/6	0.79	0.60	23,38,46,56	0
9	UYA	C	310	8/9	0.82	0.27	26,35,45,47	0
5	F8O	C	306	11/11	0.85	0.20	30,40,49,55	0
3	GOL	C	305	6/6	0.85	0.17	28,50,60,71	0
7	BU4	C	308	6/6	0.88	0.21	27,38,50,51	0
8	MV9	C	309	8/9	0.90	0.26	26,29,32,34	0
10	FH6	C	311	13/13	0.90	0.18	22,27,36,40	0
6	FDO	C	307	10/10	0.93	0.14	35,42,46,48	0
2	IMD	C	301	5/5	0.96	0.16	31,39,47,49	0
4	NI	C	304	1/1	0.96	0.05	35,35,35,35	0

The following is a graphical depiction of the model fit to experimental electron density of all instances of the Ligand of Interest. In addition, ligands with molecular weight > 250 and outliers as shown on the geometry validation Tables will also be included. Each fit is shown from different orientation to approximate a three-dimensional view.

For Manuscript Review

Electron density around UYA C 310:

$2mF_o$-DF_c (at 0.7 rmsd) in gray
mF_o-DF_c (at 3 rmsd) in purple (negative)
and green (positive)

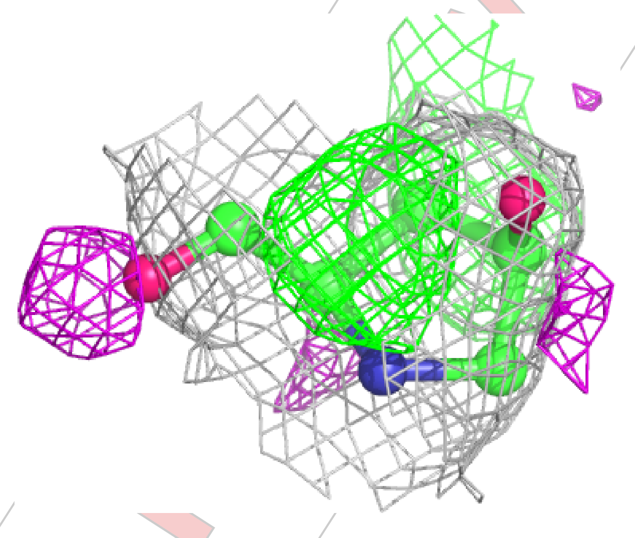

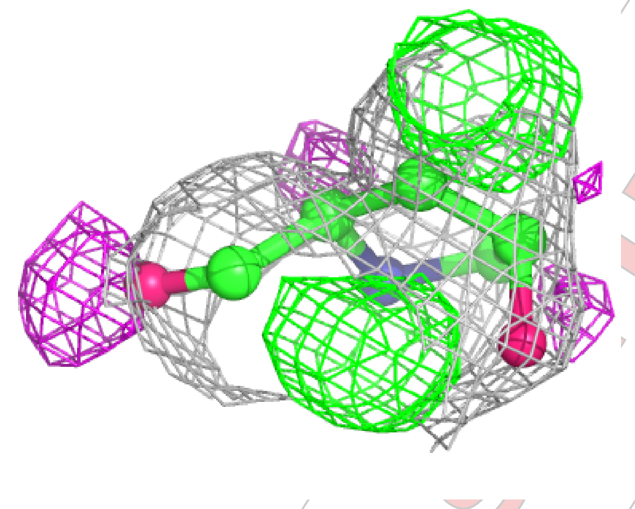

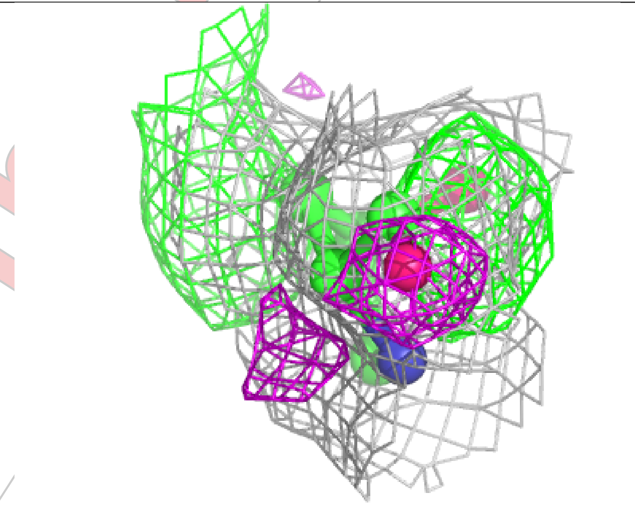

For Manuscript Review

Electron density around F8O C 306:

$2mF_o$-DF_c (at 0.7 rmsd) in gray
mF_o-DF_c (at 3 rmsd) in purple (negative)
and green (positive)

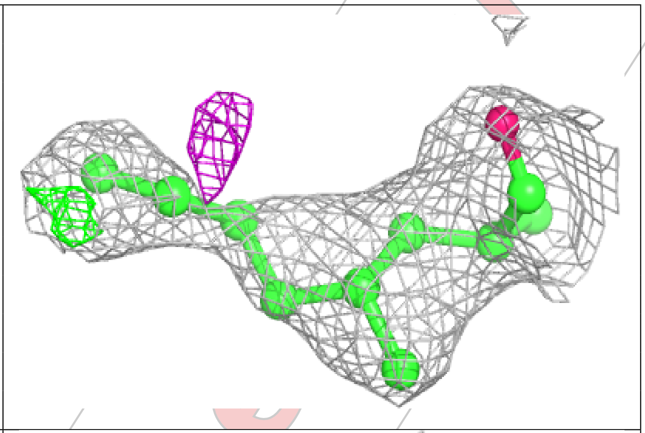

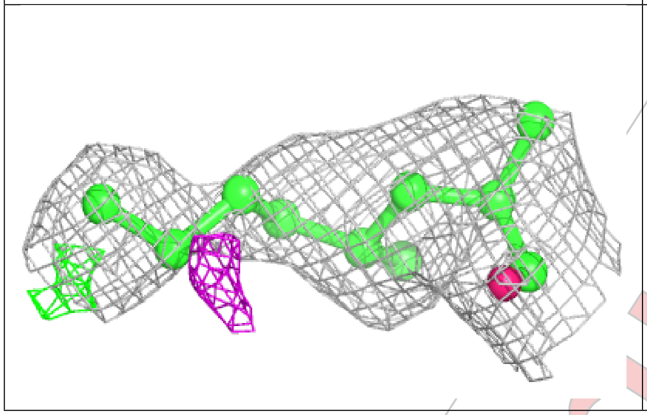

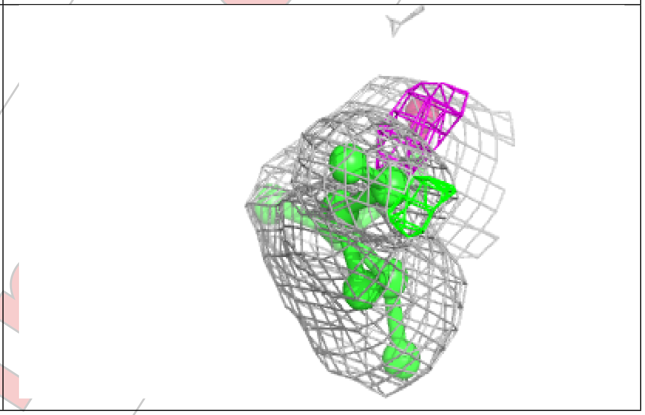

Electron density around BU4 C 308:

$2mF_o$-DF_c (at 0.7 rmsd) in gray
mF_o-DF_c (at 3 rmsd) in purple (negative)
and green (positive)

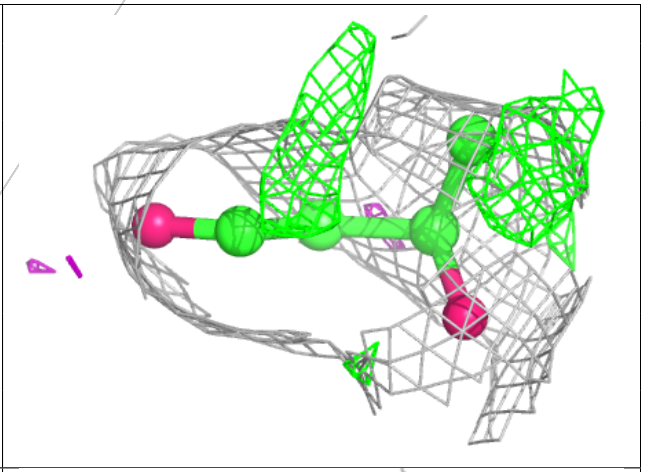

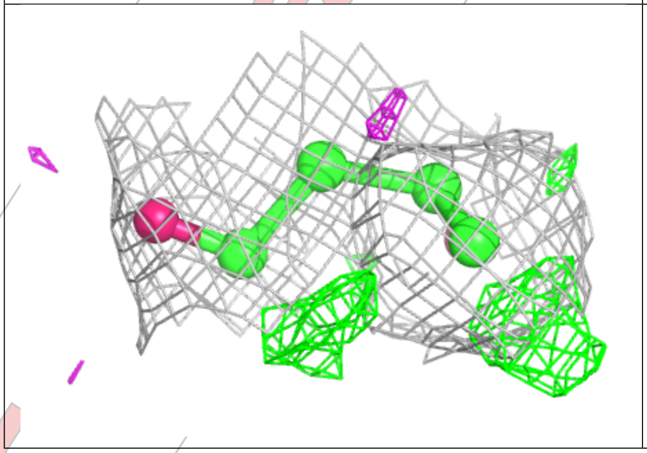

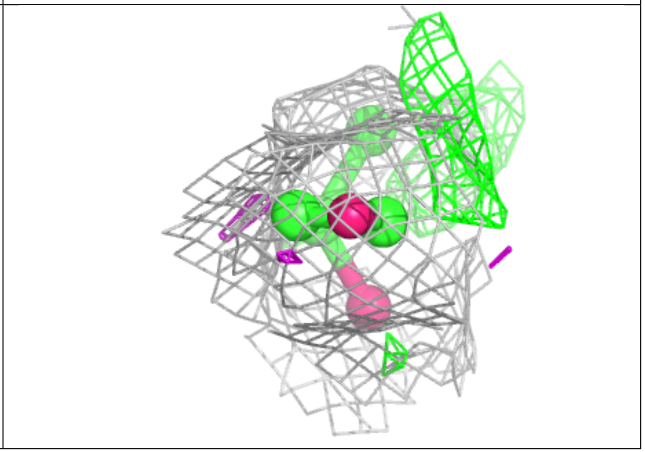

Electron density around MV9 C 309:

2mF$_o$-DF$_c$ (at 0.7 rmsd) in gray
mF$_o$-DF$_c$ (at 3 rmsd) in purple (negative)
and green (positive)

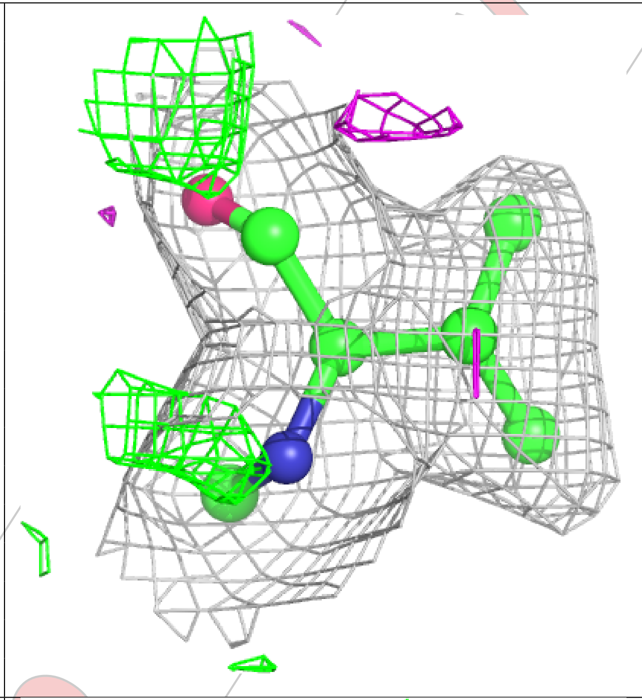

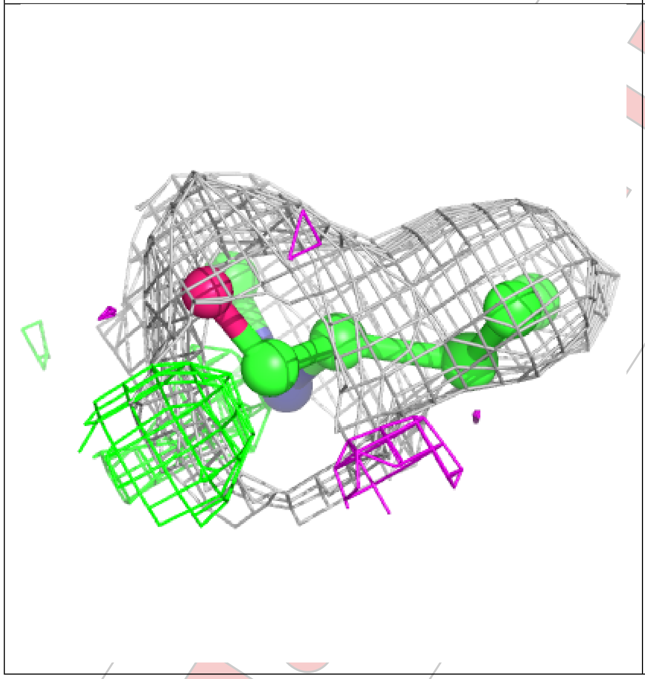

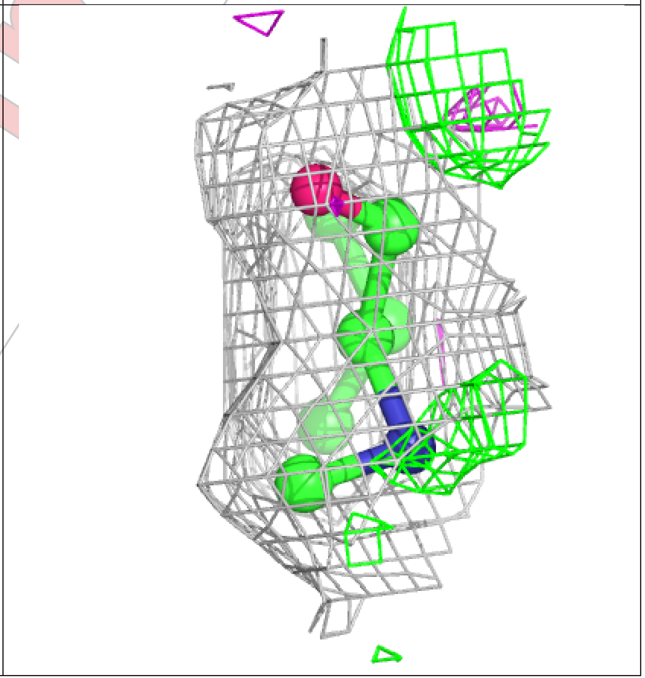

Electron density around FH6 C 311:

$2mF_o$-DF_c (at 0.7 rmsd) in gray
mF_o-DF_c (at 3 rmsd) in purple (negative)
and green (positive)

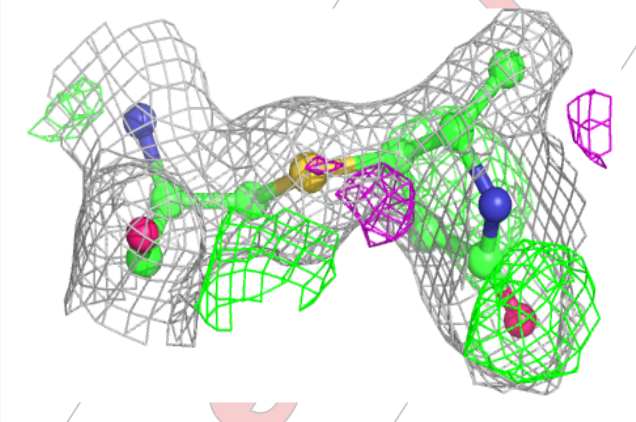

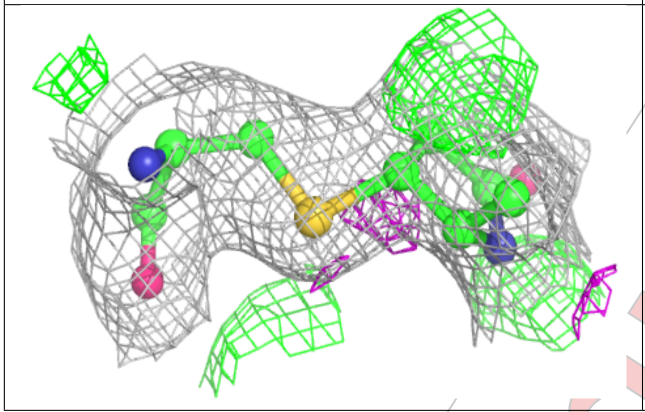

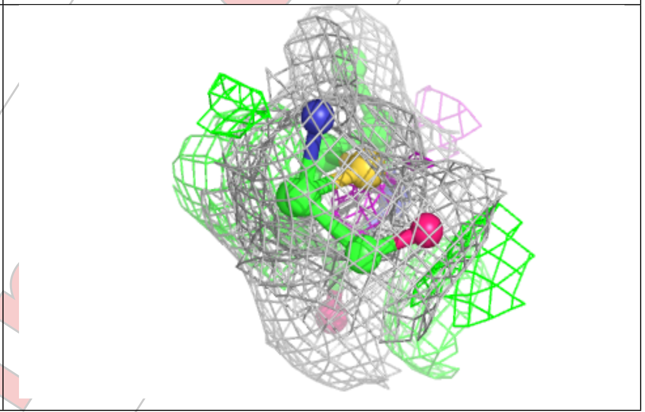

For Manuscript Review

Electron density around FDO C 307:

$2mF_o$-DF_c (at 0.7 rmsd) in gray
mF_o-DF_c (at 3 rmsd) in purple (negative)
and green (positive)

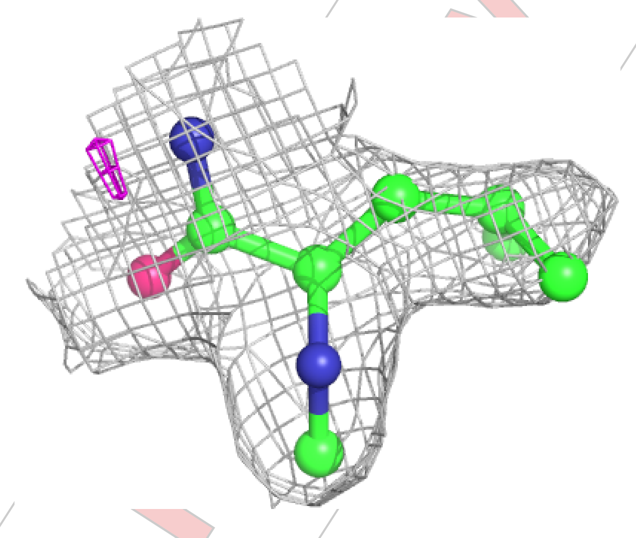

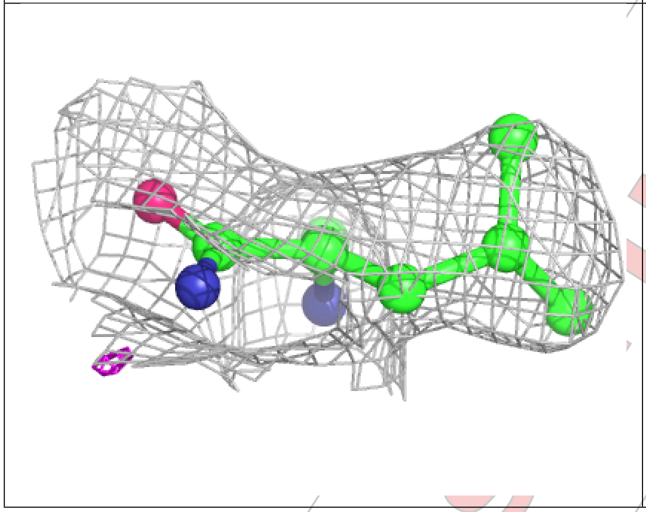

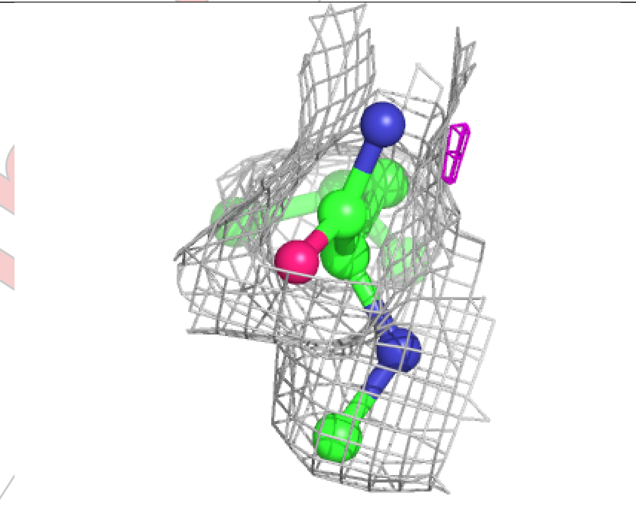

6.5 Other polymers ⓘ

There are no such residues in this entry.

For Manuscript Review
