# Supplementary material for: Non-vesicular phosphatidylinositol transfer plays critical roles in defining organelle lipid composition
Source: EMBO J. 2024 Apr 16;43(10):6. doi: 10.1038/s44318-024-00096-3 (PMC11099152; doi:10.1038/s44318-024-00096-3)
Supplement: Supplementary file 11 — Expanded View Figures [file 44318_2024_96_MOESM11_ESM.pdf]

Expanded View Figures

Figure EV1. Ability of recombinant PITPNA proteins to extract PI and PC from prelabeled membranes.

(**A**, **B**) Membranes prepared from HEK293-AT1 cells prelabeled with [^{14}C]acetate overnight were incubated with purified recombinant PITPNA wild-type or mutated in the indicated residues. After centrifugation, to pellet the membranes, the supernatant was subjected to lipid extraction and TLC analysis as detailed in the Methods. Autoradiography films with different exposure times are shown. (**B**) PI and PC spots were quantified from two independent experiments using a Phosphor-Imager and normalized to the values obtained in the wild-type proteins. (**C**) The same amounts of PITPs used in the above lipid binding assays were also run on SDS gels and visualized with Coomassie staining. (**D**, **E**) Western Blot analysis of the various mutant mRFP-tagged PITPNA and PITPNB proteins expressed in the rescue experiments. Membranes were probed with specific antibodies against PITPNA and PITPNB, which were generous gift from Dr. Shamshad Cockcroft, as indicated.

© The Author(s)

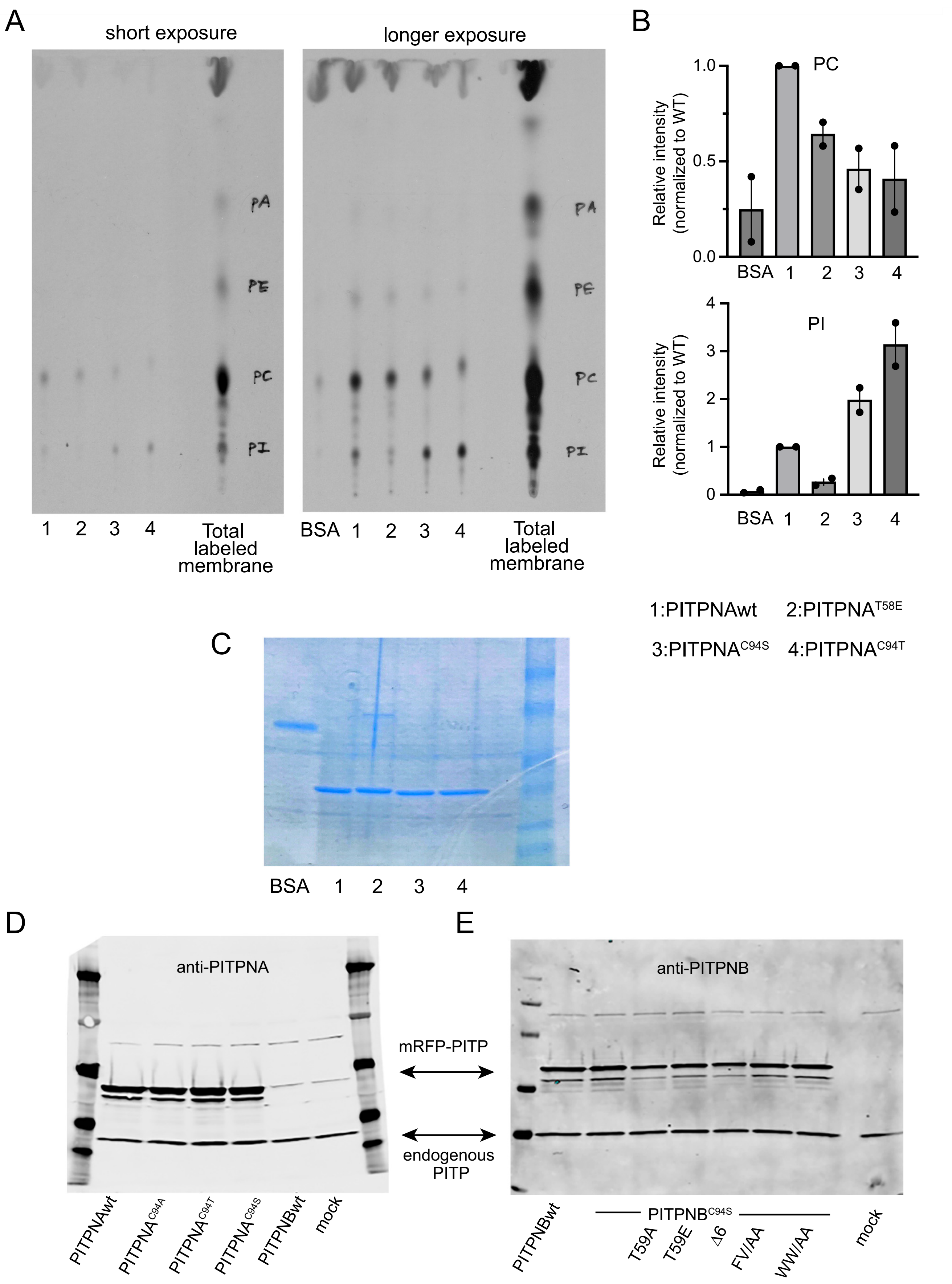
A
short exposure
longer exposure
PA
PE
PC
PI
1 2 3 4 Total labeled membrane
BSA 1 2 3 4 Total labeled membrane
B
PC
PI
Relative intensity (normalized to WT)
1.0
0.5
0.0
4
3
2
1
0
BSA 1 2 3 4
1:PITPNAwt 2:PITPNA^{T58E}
3:PITPNA^{C94S} 4:PITPNA^{C94T}
C
BSA 1 2 3 4
D
anti-PITPNA
mRFP-PITP
endogenous PITP
PITPNAwt
PITPNA^{C94A}
PITPNA^{C94T}
PITPNA^{C94S}
PITPNBwt
mock
E
anti-PITPNB
PITPNBwt
PITPNB^{C94S}
T59A
T59E
Δ6
FV/AA
WW/AA
mock

 © The Author(s)

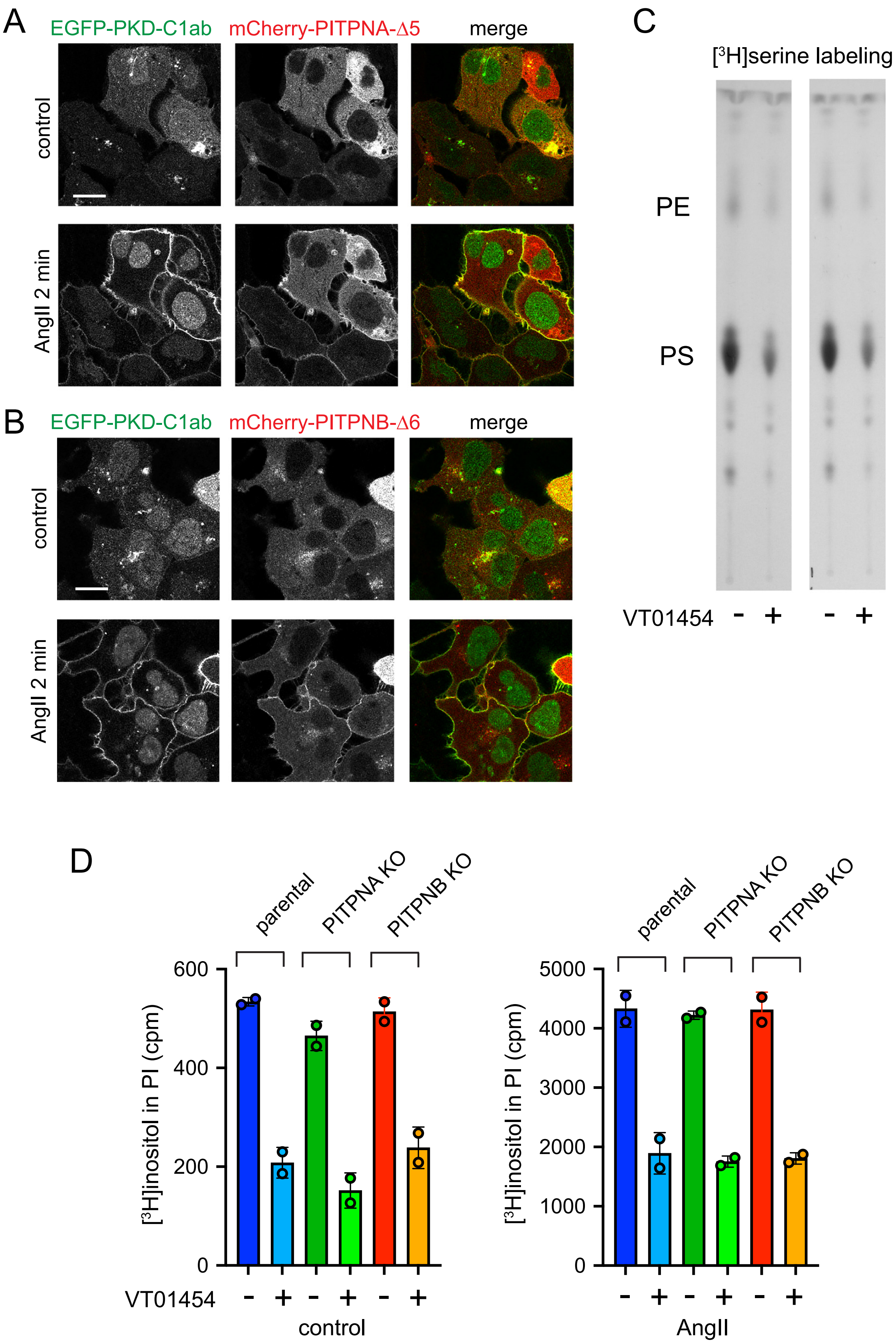
A
EGFP-PKD-C1ab
mCherry-PITPNA-Δ5
merge
control
AngII 2 min
B
EGFP-PKD-C1ab
mCherry-PITPNB-Δ6
merge
control
AngII 2 min
C
[3H]serine labeling
PE
PS
VT01454 - + - +
D
parental
PITPNA KO
PITPNB KO
[3H]inositol in PI (cpm)
600
400
200
0
VT01454 - + - + - +
control
5000
4000
3000
2000
1000
0
AngII

Figure EV2. Co-localization of C-terminally truncated PITPs and a DAG sensor in HEK293-AT1 cells and effects of VT01454 on synthetic rates of PS and PI production.

(**A**, **B**) Representative confocal images showing HEK293-AT1 cells transfected with the indicated constructs before (top rows) and after (bottom rows) stimulation with AngII (100 nM). Note the rapid increase in PM association of both the DAG sensor (PKD-C1ab) and the truncated PITPs. Scale bar 10 μm (note that all images in panels (**A**) and (**B**), respectively, show the same cells in the various channels before and after stimulation). (**C**) Incorporation of [^{14}C]-serine into cellular lipids in a 2 h incubation period shown as two biological replicates (see Methods for details). Note the inhibition of PS synthesis by treatment with VT01454 (100 nM), which was present throughout the labeling period. (**D**) Effects of VT01454 on the rate of PI labeling with *myo*-[^{3}H]inositol in a 1 h incorporation period either in the presence (right) or absence (left) of AngII. The results of two independent experiments are plotted. Parental HEK293-AT1 (blue) or their PITPNA (green) or PITPNB (red/orange) K/O derivatives are shown. Note the different scales of the two graphs.

 © The Author(s)

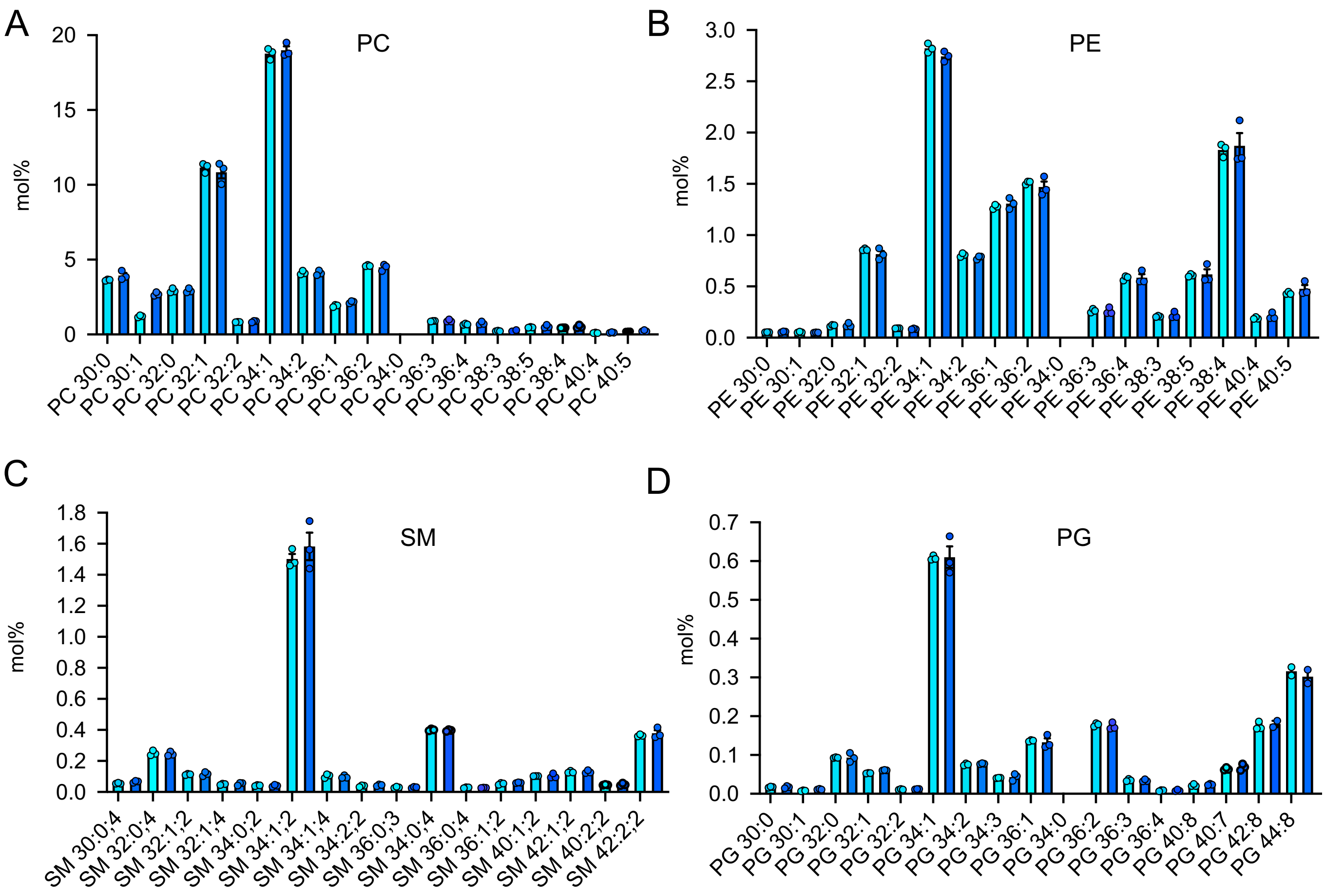

Figure EV3. Lipidomics analyses of HEK293-AT1 cells treated with VT01454.

HEK293-AT1 cells were treated with VT01454 (100 nM) or DMSO for 90 min and prepared for lipidomic analysis, which were performed by Lipotype as described in the Methods. Means ± SEM and the individual data points are shown from biological triplicates from one experiment that was repeated with essentially the same results. Dark columns show VT01454-treated cells. No significant changes were observed in either of the four lipid classes shown in panels (**A–D**).
